# The catheterized urinary tract selects for *MRR1*-mediated efflux and fluconazole resistance in *Candida albicans* biofilms

**DOI:** 10.1128/mbio.01785-26

**Published:** 2026-09-14

**Authors:** Shane A. Cristy, Jennifer N. Walker, Michael C. Lorenz

**Affiliations:** 1 Department of Microbiology and Molecular Genetics, The University of Texas Health Science Center at Houston 12340, Houston, Texas, USA; 2 Microbiology and Infectious Diseases Program, MD Anderson UTHealth Graduate School of Biomedical Sciences 12340 , Houston, Texas, USA; 3 Department of Epidemiology, School of Public Health, University of Texas Health Science Center at Houston 49219, Houston, Texas, USA; Instituto Carlos Chagas, Curitiba, Brazil

**Keywords:** urinary tract infection, *Candida albicans*, drug resistance mechanisms, biofilms

## Abstract

Catheter-associated urinary tract infections (CAUTIs) are the most common nosocomial infection in developed countries, and *Candida* species are among the most frequently isolated organisms. Despite this, little is known about the biology, host-pathogen interactions, or outcomes of these infections, and this has led to uncertain guidelines for clinical management of *Candida* CAUTIs. Here, we develop the first physiologically relevant artificial urine medium (AUM) that supports fungal growth in a manner similar to, but more consistent than, human urine samples. We demonstrate that human catheter-associated (CA) clinical isolates of *C. albicans* exhibit environment-dependent fluconazole resistance: many isolates determined to be susceptible by standard CLSI testing in RPMI (MIC ≤ 2 µg/mL) were fully resistant (MIC ≥ 128 µg/mL) when grown in pooled human urine or AUM, complicating clinical management, which is based on catheter exchange and fluconazole treatment. Transcriptomic profiling of biofilms formed in AUM revealed a remarkably convergent upregulation of efflux and detoxification processes across clinical isolates with diverse biofilm phenotypes. Whole-genome sequencing of the CA isolates identified variant alleles of transcriptional regulators of drug efflux, including *MRR1*, that have been previously associated with antifungal resistance. A competition assay confirmed that Mrr1 provides a fitness advantage in urine and AUM in a urea-dependent manner. Thus, we show that the urinary environment promotes a unique biofilm differentiation program and selects for adaptations that increase drug resistance and would be predicted to render standard treatment regimens ineffective.

**IMPORTANCE:**

Catheter-associated urinary tract infections are the most common nosocomial infection in the United States, and *Candida albicans* is one of the most frequently isolated organisms from these infections. Despite this high prevalence, few molecular studies have examined *C. albicans* biology in the urinary environment, and recommendations for clinical management lack robust evidence. Here, we show that clinical catheter-associated isolates of *C. albicans* identified as susceptible to fluconazole by standard clinical microbiology testing were resistant when grown in human or artificial urine. We identified transcriptional responses intrinsic to the urinary environment that produce this environment-specific resistance phenotype. Biofilm growth in the urinary environment induces cellular processes for efflux and detoxification. These findings suggest that standard susceptibility testing may not predict fluconazole efficacy in the urinary tract and underscore the need for niche-informed approaches to antifungal management of these common infections.

## INTRODUCTION

Catheter-associated urinary tract infections (CAUTIs) are the most common nosocomial infection in the United States (1). Approximately 20% of hospitalized patients (2, 3) and 12% of nursing home residents (4) use indwelling catheters. Catheter placement causes damage to the urothelium, resulting in chronic bladder inflammation and frequently chronic kidney inflammation (5). This damage activates the wound healing response, which releases fibrinogen (Fg) into the bladder (6). Fg deposition onto the urinary catheter provides a substrate for a broad array of microbes to attach and subsequently form biofilms (7–10). Furthermore, the incidence of microbes asymptomatically colonizing the urine increases by 3–7% per day of urinary catheterization, and can result in substantial risk of associated complications for long-term catheterized individuals, including development of symptomatic infection or other adverse outcomes such as secondary bloodstream infections or increased mortality (11–14). The long-term catheterized bladder environment also typically fosters polymicrobial communities that can exist commensally within the host or become pathogenic (15–17).

*Candida albicans* has been reported to be the second most common etiological agent of symptomatic CAUTI (behind *Escherichia coli*) (18), and a recent meta-analysis determined that *Candida* species (with *C. albicans* being the predominant member) are the most commonly isolated organisms from urinary catheters in intensive care units (19). Despite the frequency of *Candida* CAUTIs, the pathology and biology of these infections are largely unexplored (20), and treatment recommendations—which often withhold antifungal therapy—lack robust evidence (21). The current guidelines for managing a CAUTI caused by *C. albicans* are to remove the catheter if possible and treat with fluconazole (depending on organism susceptibility as determined by standard CLSI testing) or amphotericin B deoxycholate (21). For non-healthcare-associated *C. albicans* strains, fluconazole resistance is low (22, 23), but fluconazole prophylaxis selects for resistant strains of *C. albicans* or other species that are intrinsically less susceptible, such as *Nakaseomyces glabratus* (formerly *Candida glabrata*) (24, 25). Resistance to fluconazole can be caused by a variety of genetic mutations that include structural alterations to the target enzyme of the drug (Erg11), increased production of the target enzyme, and increased efflux activity to pump the drug out of the cell (26).

In addition to genetic changes, developmental programs such as biofilm formation can promote antimicrobial tolerance (27, 28). Efflux pump activity, the extracellular matrix, and the presence of quiescent persister cells all reduce the efficacy of antifungal drugs against *Candida* biofilms (27, 28). These mechanisms have been well described in *in vitro* biofilms across a range of abiotic substrates and media conditions (29–31), as well as *in vivo* in the context of biofilms on denture material (32) and central venous catheters (33). A study of *C. albicans* CAUTI in rats demonstrated that the urinary catheter fungal burden of a fluconazole susceptible strain, K1, was only minimally altered after fluconazole or amphotericin B deoxycholate treatment (34); thus, demonstrating antifungal tolerance in CAUTI biofilms *in vivo*. Yet, the molecular mechanisms underlying this phenomenon are virtually unknown.

The *C. albicans* biofilm transcriptome has been examined in various media environments and substrates (35), but only one study has been conducted in the urinary environment (7). Notably, human urine varies significantly in composition between individuals and within an individual over time, which presents challenges to reproducibility (36, 37). This is only partially mitigated by pooling urine from multiple donors (36, 37). Standardized urine can be purchased commercially, but is still pooled from human donors, and storage and shipping conditions, time from collection, exposure to heat and oxidation introduce additional variables. Any of these options are subject to variation that complicates replication between, or even within, labs. To overcome reproducibility issues, several formulations of artificial urine have been developed for bacterial growth, but most do not support *Candida* growth (38, 39). For the formulations that do support *Candida* growth, they contain non-physiological concentrations of key metabolites, especially glucose (40), making the relevance of the results questionable. For this study, we developed the first physiologically relevant synthetic artificial urine medium (AUM) that supports *C. albicans* growth in planktonic and biofilm conditions. This AUM builds off of a recently published defined base formula (41) to which physiologically relevant concentrations of glucose, small peptides, and several micronutrients are added.

For this work, we leveraged this AUM, human urine, and a panel of clinical catheter-associated (CA) *C. albicans* isolates previously collected from long-term catheterized individuals (15, 16, 42) to characterize genetic, transcriptomic, and phenotypic determinants of *Candida* adaptation to the urinary environment. We found significant differences in the biofilm biomass potential between isolates in an *in vitro* urinary catheter environment. Despite these differences, we found striking similarities in the transcriptomes between the lab strain SC5314 and CA isolates in urinary biofilms. One shared attribute was the upregulation of efflux mechanisms and genes that respond to chemical stressors. Antifungal susceptibility testing demonstrated that the majority of our CA clinical isolates were susceptible to fluconazole in the standard CLSI assay (43), but completely resistant in urine or AUM, suggesting that fluconazole may be a suboptimal choice for therapy.

Whole-genome sequencing of CA clinical isolates revealed multiple putative gain-of-function mutations in key efflux pump regulator genes, including *MRR1*, which has previously been associated with increases in azole resistance in clinical isolates of *C. albicans* (44) and *C. lusitaniae* (45). We found that the Mrr1 regulon is enriched in the set of genes that are differentially expressed in AUM biofilms compared to standard biofilm growth medium. Furthermore, competition assays with an *mrr1*Δ/Δ deletion mutant confirmed the functional importance of Mrr1-regulated efflux in urinary adaptation. Finally, antimicrobial susceptibility testing of CA clinical isolates of other species suggests these traits may be common to the close relative species of *C. albicans* in the CUG clade.

## RESULTS

### A novel artificial urine medium that supports *Candida albicans* growth

**Fig 1:**
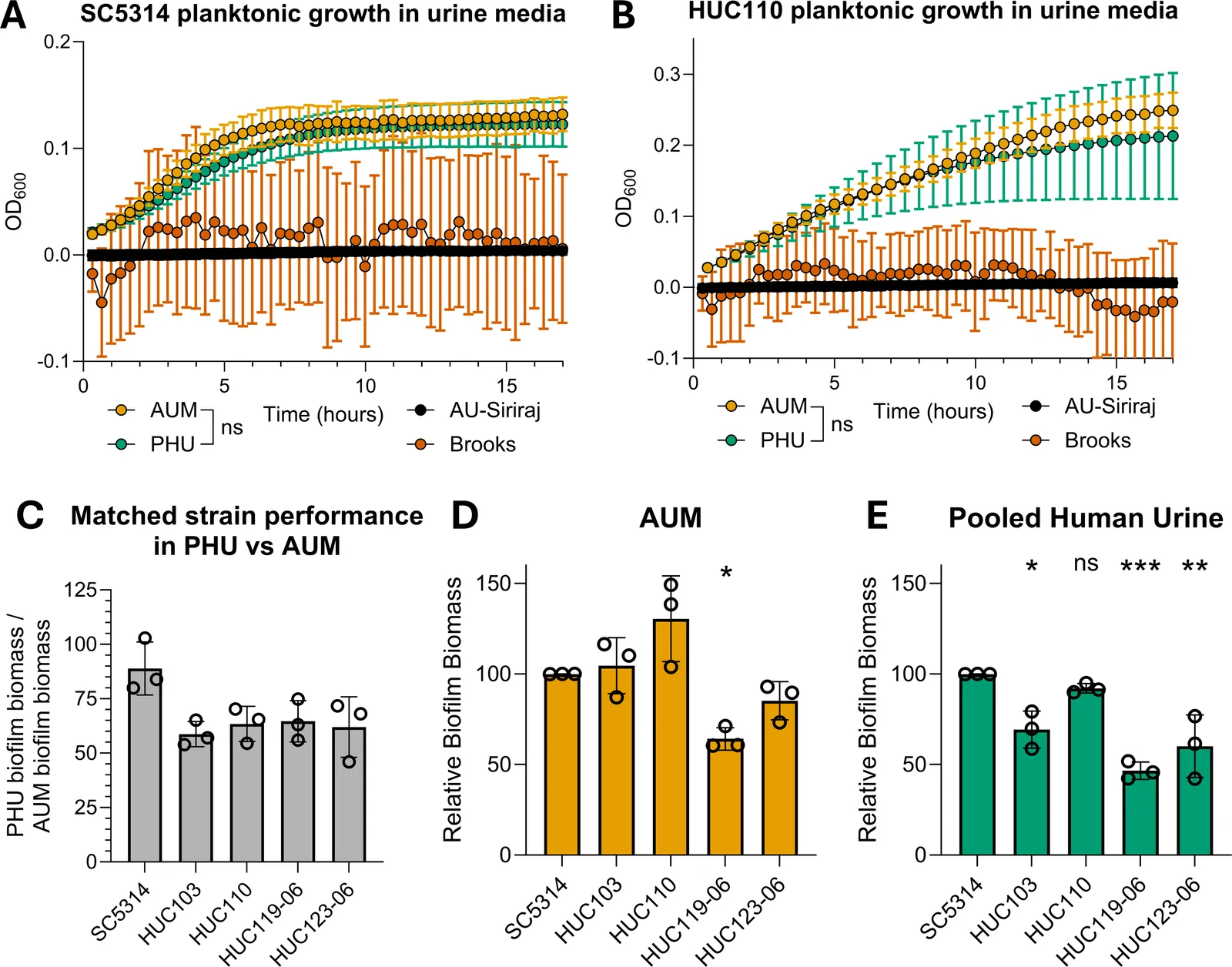
A novel artificial urine medium (AUM) that supports *Candida albicans* growth. (**A**) Planktonic growth of *Candida albicans* SC5314 measured by absorbance at 600 nm (OD_600_) in AUM, pooled human urine (PHU), AU-Siriraj, and Brooks media. (**B**) Planktonic growth of *C. albicans* HUC110 in AUM, PHU, AU-Siriraj, and Brooks media. Area under the curve was calculated for both experiments (**A and B**) and compared with a one-way analysis of variance (ANOVA) test with Sidak’s multiple comparisons test; ns, *P* value ≥ 0.05. (**C**) Comparison of biofilm formation of each strain in PHU and AUM. (**D**) Comparison of biofilm formation of each strain in AUM relative to SC5314. (**E**) Comparison of biofilm formation of each strain in PHU relative to SC5314. For panels D and E, comparisons were measured with a one-way ANOVA with Dunnett’s multiple comparisons test. Adjusted *P* values: *P < 0.05, **P < 0.01, and ***P < 0.001. ns, not significant (*P* > 0.05).

We tested several of the previously published formulations of artificial urine (38, 39, 41) for their ability to support *Candida* growth in an iterative process. We compared these artificial media to pooled human urine (PHU). Our final AUM formulation (Table 1) supported planktonic growth of *C. albicans* SC5314 better than the published Brooks (38) and AU-Siriraj (39) media, and AUM recapitulated the growth phenotype of SC5314 in PHU (Fig. 1A). Furthermore, AUM supports growth of fungal, gram-negative, and gram-positive CAUTI pathogens (Fig. S1A and B). We tested clinical CA isolates of *C. albicans* for planktonic growth in AUM and PHU, and we found that these strains also demonstrate similar growth dynamics in both media (Fig. 1B; Fig. S1C), but typically display more variability in PHU than AUM. Some of these CA isolates, such as HUC110, grew better than SC5314 in the urinary environment (Fig. 1B; Fig. S1D).

**TABLE 1:** Artificial urine medium formulation

Chemical compound	Brooks AUM (38) (mmol/L)	AU-Siriraj (39) (mmol/L)	Sarigul AUM (41) (mmol/L)	Cristy AUM (mmol/L)	Cristy AUM (g/L)
Na_2_SO_4_	10.000	9.000	11.965	11.965	1.700
Uric acid	0.400	1.000	1.487	1.487	0.250
Sodium citrate·2H_2_O	2.000	5.000	2.450	2.450	0.720
Creatinine	7.000	4.000	7.791	7.791	0.881
Urea	170.000	200.000	249.750	249.750	15.000
KCl		30.000	30.953	30.953	2.308
NaCl	90.000	54.000	30.053	30.053	1.756
CaCl_2_	2.500	3.000	1.166	1.166	0.185
NH_4_Cl	25.000	15.000	23.667	23.667	1.266
Potassium oxalate·H_2_O			0.190	0.190	0.035
MgSO_4_·7H_2_O	2.000	2.000	4.389	4.389	1.082
NaH_2_PO_4_ (monobasic)		3.600	18.667	18.667	2.912
Na_2_HPO_4_ (dibasic)		0.400	4.667	4.667	0.831
Tryptone				0.909	0.100
Yeast nitrogen base				various	1.700
Glucose				0.833	0.150
Biotin				82 nmol/L	22 µg/L
Peptone	1.000 g/L				
Yeast extract	0.005 g/L				
Lactic acid	1.100				
NaHCO_3_	25.000				
FeSO_4_ (ferrous sulfate)	0.005				
KH_2_PO_4_ (monobasic)	7.000				
K_2_HPO_4_ (dibasic)	7.000				
Sodium oxalate		0.100			

Given the difference in planktonic growth of SC5314 and HUC110 in AUM (Fig. 1A and B; Fig. S1C), we sought to establish whether the historical lab strain SC5314, originally isolated from a patient with disseminated candidiasis (46), is a suitable model strain for CAUTI. We compared SC5314 and several CA isolates in a standard crystal violet biofilm assay grown on Fg-coated 96-well polystyrene plates in both PHU and AUM. We found that AUM supported stronger biofilm growth than PHU across all strains tested, but this disparity was lowest with SC5314 (Fig. 1C). We also compared the biofilm biomass between strains grown in either AUM or PHU (Fig. 1D and E). Biofilm formation for the CA isolates was either equivalent to or lower than SC5314, and, after accounting for the stronger biofilm growth of the CA isolates having stronger in AUM than PHU, the pattern of relative biofilm formation across strains was generally similar in the two media. Overall, these data demonstrate that (i) SC5314 is a reasonable model strain for CAUTI biofilm and growth in urine, comparable to most CA isolates, (ii) that strains with growth advantages in the urinary environment like HUC110 exist, and that (iii) AUM is an accurate model for PHU but is less variable and more standardized.

### Catheter-associated clinical isolates display variable biofilm phenotypes in a novel CAUTI *in vitro* model

Previous publications have detailed murine *in vivo* CAUTI models (7, 34), glass bladder *in vitro* models (47), and other 96-well plate *in vitro* models (48). We sought to develop an *in vitro* catheter model that more closely represents CAUTI, could be used in high throughput, accounts for the silicone substrate of typical urinary catheters, and recapitulates the biofilm growth observed from clinical samples of urinary catheters (42). To do so, we used sections of human silicone urinary catheters coated with Fg as the substrate for our *in vitro* CAUTI biofilm model.

**Fig 2:**
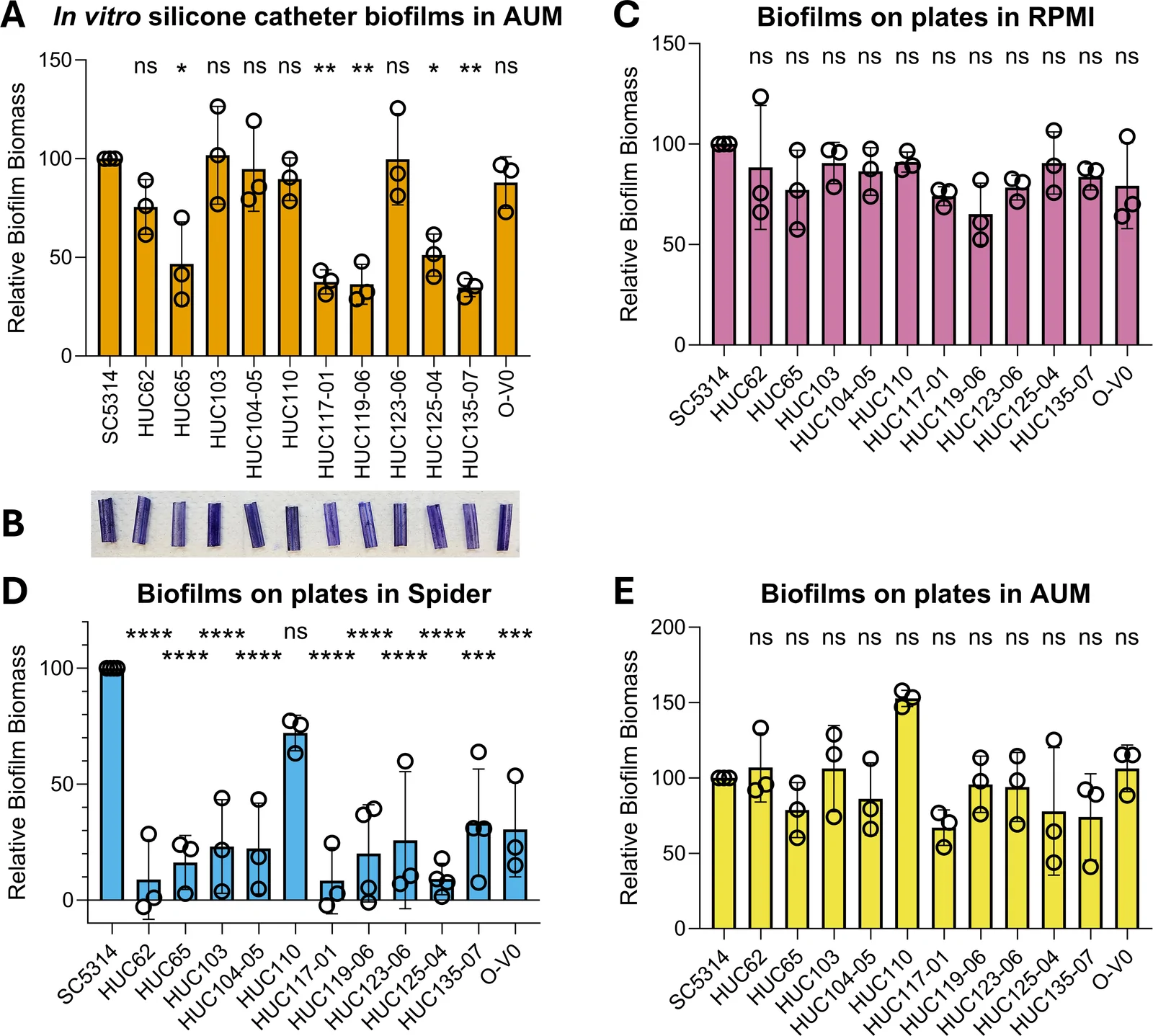
Catheter-associated clinical isolates display variable biofilm phenotypes. (**A**) Biofilm formation of CA isolates on silicone foley catheters in AUM relative to SC5314. (**B**) Representative catheters from (**A**) stained with crystal violet arranged in the same order. (**C–E**) Biofilm formation of CA isolates on 96-well polystyrene plates relative to SC5314 in (**C**) RPMI, (**D**) Spider medium, and (**E**) AUM. Comparisons were measured with a one-way ANOVA with Dunnett’s multiple comparisons test. Adjusted *P* values: *P < 0.05, **P < 0.01, ***P < 0.001, and ****P < 0.0001. ns, not significant (*P* > 0.05).

We selected single CA isolates from 11 different individuals (15, 16, 42)—excluding any longitudinal isolates to avoid biasing our results with multiple isolates of the same strain—and assessed biofilm biomass formed on the silicone catheters in AUM relative to SC5314 (Fig. 2A). Representative images of the catheters stained with crystal violet are shown in Fig. 2B. Based on this, we sorted the CA isolates into two broad groups—one group with biofilm formation comparable to SC5314, including HUC110, and one group with reduced biofilm formation including HUC119-06. Together, these data demonstrate that SC5314 is an appropriate model and genetic platform for CAUTI molecular studies, as it groups with CA isolates that form robust biofilm.

### Biofilm differentiation is highly dependent on environmental conditions

We considered that the differences in biofilm formation we observed in the *in vitro* catheter assay could be attributable to the general biofilm formation habits of these strains, so we next tested the CA isolates in the more standard biofilm assays using RPMI (Fig. 2C) and Spider (Fig. 2D) media on 96-well polystyrene plates. We also grew biofilms on Fg-coated plates in AUM (Fig. 2E). RPMI medium is a strong inducer of biofilm formation (49), and in this environment, all the CA isolates formed robust biofilms comparable to SC5314, with only modest variation between strains; the most attenuated strain, HUC119-06, was only 35% reduced relative to SC5314 (Fig. 2C). Spider medium is distinct for being a non-physiological minimal medium, and a very strong inducer of biofilm formation (50), especially in SC5314 (51). In this environment, HUC110 was the only isolate not significantly different from SC5314 (Fig. 2D). Conversely, in the AUM Fg-coated plate assay, all strains formed robust biofilms similar to SC5314 (Fig. 2E). Both Spider and AUM are nutrient-limited media, but with very different compositions, and these data reinforce that biofilm differentiation programs depend on environmental conditions and that individual strains can be adapted for one set of conditions vs others. These experiments give us a framework with which to consider the CA isolates as grouping into two distinct phenotypic groups, with HUC110 and HUC119-06 being the most extreme examples of each group.

### Transcriptional profiling of SC5314, HUC110, and HUC119-06 biofilms

**Fig 3:**
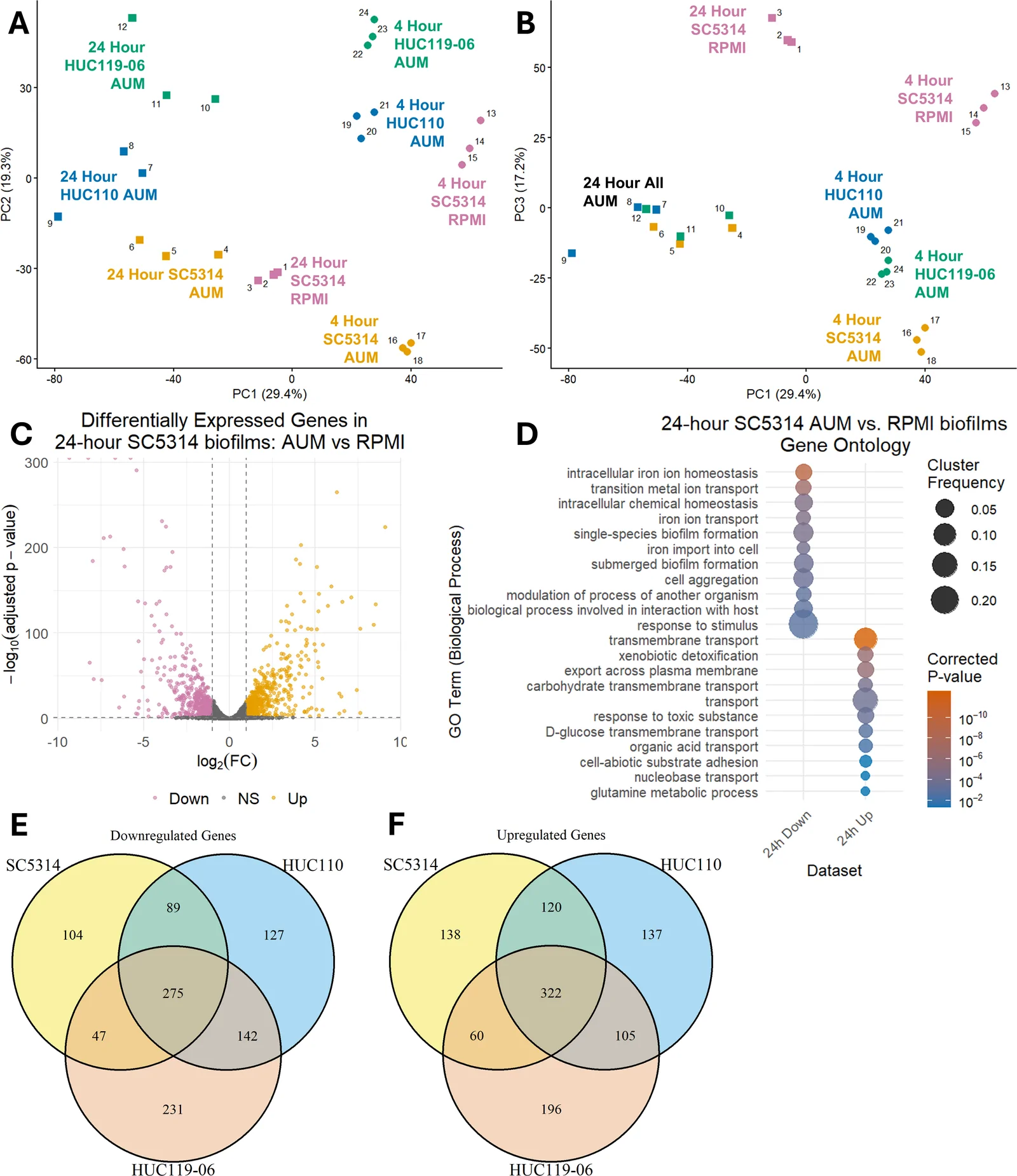
The unique artificial urine medium (AUM) transcriptome. (**A and B**) Principal component analysis of gene expression data from biofilms of SC5314, HUC110, and HUC119-06 grown in AUM and RPMI (SC5314 only) at 4-h and 24-h time points. (**C**) 1,155 genes were differentially expressed in 24-h biofilms of SC5314 in AUM (Upregulated) vs. RPMI (Downregulated). (**D**) Gene ontology analysis of significantly different processes in SC5314 in AUM (vs RPMI) at 24 h. (**E and F**) Comparison of genes significantly (**E**) downregulated or (**F**) upregulated in SC5314, HUC110, and HUC119-06 in AUM at 24 (all vs SC5314 in RPMI at 24 h).

To understand the transcriptional basis of biofilm formation in the catheterized urinary tract, we profiled the transcriptomes via mRNA sequencing of young (4-h) and mature (24-h) biofilms of SC5314, HUC110, and HUC119-06 grown in AUM on Fg-coated polystyrene plates. As a control and comparison, we also included SC5314 biofilms grown in RPMI at 4-h and 24-h time points. Principal component analysis (PCA) confirmed the reproducibility of the transcript profiles as all replicates per strain and time point clustered together (Fig. 3A). There was also clear separation between the 4-h and 24-h time points in principal component 1 (PC1). In contrast, PC2 generally represented strain-based variability. The first two principal components accounted for less than half of the total variation in the data (29.4% and 19.3%, respectively), so we expanded our view to the third principal component, which describes 17.2% of the data set variation. This dimension of the data clearly sorts all the RPMI samples from all the AUM samples, but more interestingly, it sorts all the 24-h AUM samples together (Fig. 3B). This indicates a remarkable convergence of responses across genotypically and phenotypically divergent strains that contribute to growth and success in the catheterized bladder environment.

We examined the differentially expressed genes between these sample groups to understand the different dimensions of how biofilms grow in the catheterized urinary tract environment and what makes them unique compared to other well-studied biofilm niches. First, we examined the differences between SC5314 grown in AUM and RPMI. There were 1,740 differentially expressed genes in the early (4 h) biofilms (Fig. S2A; Table S2). Gene ontology (GO) term analysis determined that within the AUM biofilms, processes involved in metabolism of amino acids and carboxylic acids were upregulated, while processes involved in the transport and metabolism of carbohydrates were downregulated (Fig. S2B), presumably reflective of the considerable nutritional differences between these media: RPMI contains more glucose than AUM, and the amino acid metabolic processes could be an indication that SC5314 is using the Fg on the substrate surface as an alternative carbon source. There were 1,155 genes differentially expressed between the 24-h AUM and RPMI biofilms (Fig. 3C; Table S2). Metabolic processes were no longer the major differences identified in the GO analysis for these mature biofilms, indicating that by this time, both biofilms have likely consumed the vast majority of key nutrients. In both the 4-h and 24-h AUM biofilms, processes involving iron homeostasis were downregulated, but this signal was much stronger in the 24-h biofilms. Interestingly, within mature SC5314 biofilms, several processes related to export and detoxification were upregulated (Fig. 3D). There were 20 upregulated genes annotated within the GO term for “xenobiotic detoxification for membrane transport,” including *MDR1*, *CDR3*, *QDR2*, *NAG3*, *NAG4*, and *FLU1*. Each of these is a known or predicted drug efflux pump and has been associated with altered susceptibility to fluconazole, cycloheximide, nitroquinolone, and other agents with antifungal activity (52–57). This suggests that the urinary environment induces systems to export potentially toxic compounds and that this might alter susceptibility to antifungal drugs in clinical use.

Next, we compared the two clinical CA strains grown in AUM against the SC5314 RPMI-grown biofilms. After determining differentially expressed genes for each comparison, genes were sorted to identify commonalities and differences between each comparison. For the 4-h time point, there were 306 genes commonly upregulated by SC5314, HUC110, and HUC119-06 in AUM vs the SC5314 RPMI control (Fig. S2C). Once again, many of these genes were involved in metabolic processes, but “nitrogen homeostasis,” “nitrogen utilization,” and “amide transport” were among the top GO terms indicated for the intersection of all three strains. There is a high concentration of urea in urine and in AUM, which likely induces the transcription of the urea amidolyase *DUR1,2*, the urea transporter *DUR3*, and the other genes in this group (58, 59). However, loss of *DUR1*, *DUR2*, or *DUR3* did not result in a fitness defect for SC5314 in AUM (data not shown), suggesting that urea is not a primary nutrient in these conditions. All three strains shared 185 genes commonly downregulated at the 4-h time point (Fig. S2D). Common processes included heavy metal homeostasis and the metabolic shifts we observed in SC5314. These trends persisted to the 24-h time point, where 275 genes were commonly downregulated across all three strains, and many of the most downregulated processes identified by GO involve heavy metal homeostasis, which is reflective of the higher metal, especially iron, content in AUM relative to RPMI (Fig. 3E).

There were 322 genes commonly upregulated across all three strains in the 24-h mature biofilms (Fig. 3F). Many of the upregulated cell processes were related to detoxification, transmembrane transport, and metabolism, generally matching what was observed with SC5314 alone. Fully half (50.3%) of the genes upregulated in SC5314 at 24 h are also upregulated in both HUC110 and HUC119-06, a remarkable regulatory conservation given the considerable phenotypic differences between the three strains (Fig. 1D and E and 2A and DFig. 2; Fig. S1C). Several of the genes annotated under “xenobiotic detoxification by transmembrane transport,” including *QDR2*, *NAG3*, *NAG4*, and *FLU1*, were similarly upregulated across all three strains. In summary, we discovered that in the early stages of biofilm development, nutritional stresses drive the transcriptional differences. As these biofilms age, however, they converge on a similar transcriptional program that upregulates efflux pumps. This transcriptional response is similar to what is expected in response to drug treatment (44, 60, 61), despite no drug being present. These data suggest there are factors, such as the high concentration of urea and salts within AUM, that induce a general fungal stress response.

### *C. albicans* CA clinical isolates are genetically diverse

To complement the transcriptional changes that facilitate growth in urinary biofilms, we asked whether certain genotypes or genetic adaptations might promote fitness in the urinary environment. To address this question, we analyzed the genomes of our panel of 11 CA isolates from unique individuals and placed them within a maximum likelihood phylogeny constructed using a set of genomes representing the major clades of *C. albicans* from references 62, 62 (Fig. S3A). The final filtered data set contained 356,389 biallelic SNPs across 53 samples. The CA isolates were dispersed throughout the phylogeny, indicating fitness in the urinary tract is not a feature of specific genetic clades.

For study participants who had persistent *C. albicans* colonization (HUC123 and HUC135), only the terminal isolate sequence was used for the complete analysis. All four isolates from participant HUC123 were sequenced, and as has been shown in persistent colonization by bacterial species in this same patient cohort (63), longitudinal isolates from the same individual were closely related (Fig. S3B). Despite there being a catheter exchange interval at which no fungi were isolated from this participant (HUC123-05), these results are most consistent with a model that HUC123-06 is from the same closely related population as the previous isolates and not a novel colonization event.

### Catheter-associated clinical isolates encode variant alleles that are associated with antifungal resistance

We next examined the whole-genome sequencing data of the 11 CA isolates to identify variant alleles in key antifungal resistance genes that have been previously associated with drug resistance. These allele variants would have arisen, or at least have been maintained, in the absence of selective pressure from antifungal drugs, as none of the individuals enrolled in the sample collection received antifungals for at least 1 year prior (15, 16, 42).

**Fig 4:**
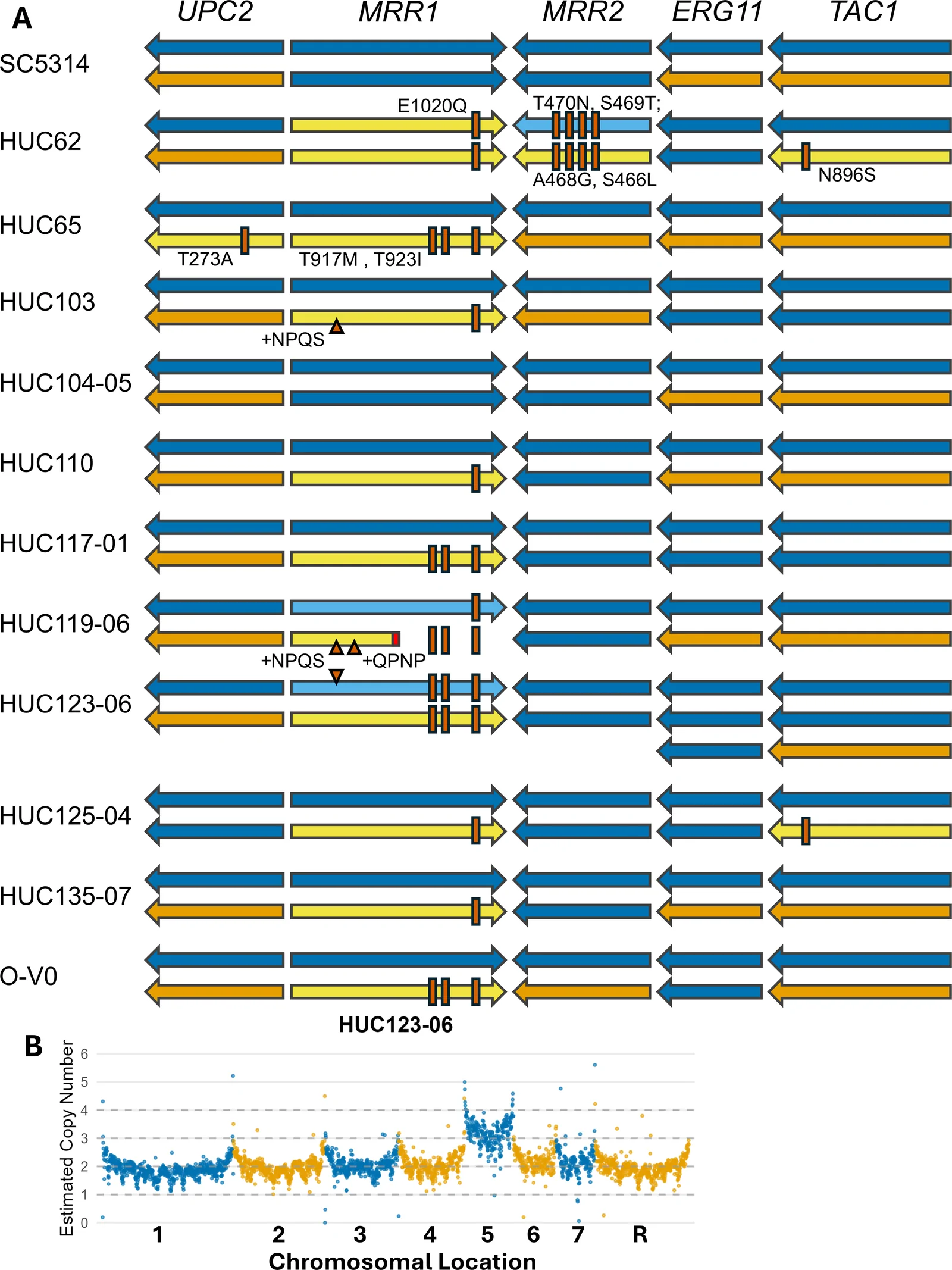
Genomes of catheter-associated clinical isolates contain variant alleles that promote antifungal resistance. (**A**) Whole-genome sequencing was performed on all clinical isolates, and key antifungal resistance genes were analyzed for variant sequences previously associated with antifungal resistance. Arrows represent genes, and different colors (blue and orange) represent alleles with distinct sequences. Lighter color alleles (yellow or cyan) represent sequences identified with antifungal resistance-associated variants. Red boxes indicate the position of specific amino acid changes that have been associated with, but either not tested or insufficient, to cause fluconazole resistance. The *MRR1* B allele of HUC119-06 has an early stop codon represented by a bright red box that interrupts the gene. (**B**) Genome coverage of WGS reads from HUC123-06 binned into 5 kb segments indicates that this strain is aneuploid with a stable trisomy of chromosome 5.

All but one of our CA isolates had at least one allele of *MRR1* that encodes glutamine at position 1,020 instead of a glutamic acid (E1020Q) (Fig. 4A). This mutation occurs in the inhibition domain of the protein (64) and is enriched in fluconazole-resistant isolates (44, 65, 66). Two additional mutations, adjacent to the inhibition domain, T917M and T923I, were present in five of the isolates. These three mutations occurring together have been previously reported in fluconazole-resistant strains isolated from other types of infections (65).

We found several strains with particularly remarkable sequences. HUC123-06 has all three of these nonsynonymous mutations (T917M, T923I, and E1020Q) on both alleles of *MRR1*, but those alleles have distinct peptide sequences due to one allele having an insertion of four residues after position 168, a natural polymorphism in *MRR1* that does not affect activity (44). Three other isolates (HUC65, HUC117-1, and O-V0) in this lineage have the same polymorphisms (Fig. 4A; Fig. S3). This suggests that this locus is under substantial evolutionary pressure in the urinary tract environment. HUC119-06 has a nonsynonymous mutation resulting in an early STOP codon in one allele of *MRR1*. This is represented here on the B allele, but with short-read sequencing alone, we were unable to determine with high confidence if the truncated allele was the one with the T917M and T923I substitutions or not.

When we expanded our investigation to other transcriptional regulators, we found additional variant alleles associated with antifungal resistance in our clinical CA isolate set (Fig. 4A). HUC65 has a substitution in *UPC2* (T273A) that was identified in resistant isolates with increased expression of *ERG11* (66). HUC62 had four mutations on each of two heterozygous alleles for *MRR2*, another regulator of efflux pumps. These four mutations—S466L, A468G, S469T, and T470N—have each been shown in isolation to confer a slight increase in fluconazole MIC (67). Finally, two CA isolates contained variant alleles of *TAC1*, the regulator of the efflux pump genes *CDR1* and *CDR2* (61). HUC62 and HUC125-04 both had the same N896S nonsynonymous mutation in *TAC1,* which lies within the activation domain of the protein. The N896S allele has been found in fluconazole-resistant isolates (66). We note that other nonsynonymous substitutions in the Tac1 activation domain have been found to be sufficient for fluconazole resistance (68, 69).

Finally, it is worth noting that HUC123-06 is aneuploid with a trisomy for chromosome 5 (Fig. 4B). *C. albicans* can tolerate stable aneuploidies that persist after they have been selected for by an environmental stressor. Key antifungal resistance genes are located on chromosome 5, including *TAC1* and *ERG11* (the target of azole drugs). Increasing the number of copies of chromosome 5 is a means by which *C. albicans* can become resistant to azole drugs, and this has been identified in clinical isolates (70). Thus, our genomic analysis identified several mechanisms, most commonly variant alleles of *MRR1*, through which these CA clinical isolates may have adapted to the urinary tract environment.

### *C. albicans* uses the Mrr1 regulon to adapt to the urinary environment

The accumulation of variant alleles of *MRR1* found in the majority of these CA isolates despite no recent history of antifungal therapy is reminiscent of *Clavispora lusitaniae* (formerly known as *Candida lusitaniae*) isolates from the lungs of cystic fibrosis patients, in which variation in fluconazole resistance in isolates from individual patients was linked to gain-of-function mutations in *MRR1* (45, 71). Mrr1 is a transcription factor that regulates several of the efflux pumps induced in our transcriptional data. We hypothesized that the Mrr1 regulon is important for adaptation to the urinary tract environment. The binding sites of Mrr1 across the *C. albicans* genome have been previously identified using chromatin immunoprecipitation (ChIP)-chip analysis (72). Approximately 450 verified ORFs are bound by Mrr1, but of those only 40 were characterized as being directly activated by a hyperactive variant of Mrr1 (Mrr1^P683S^) (72).

**Fig 5:**
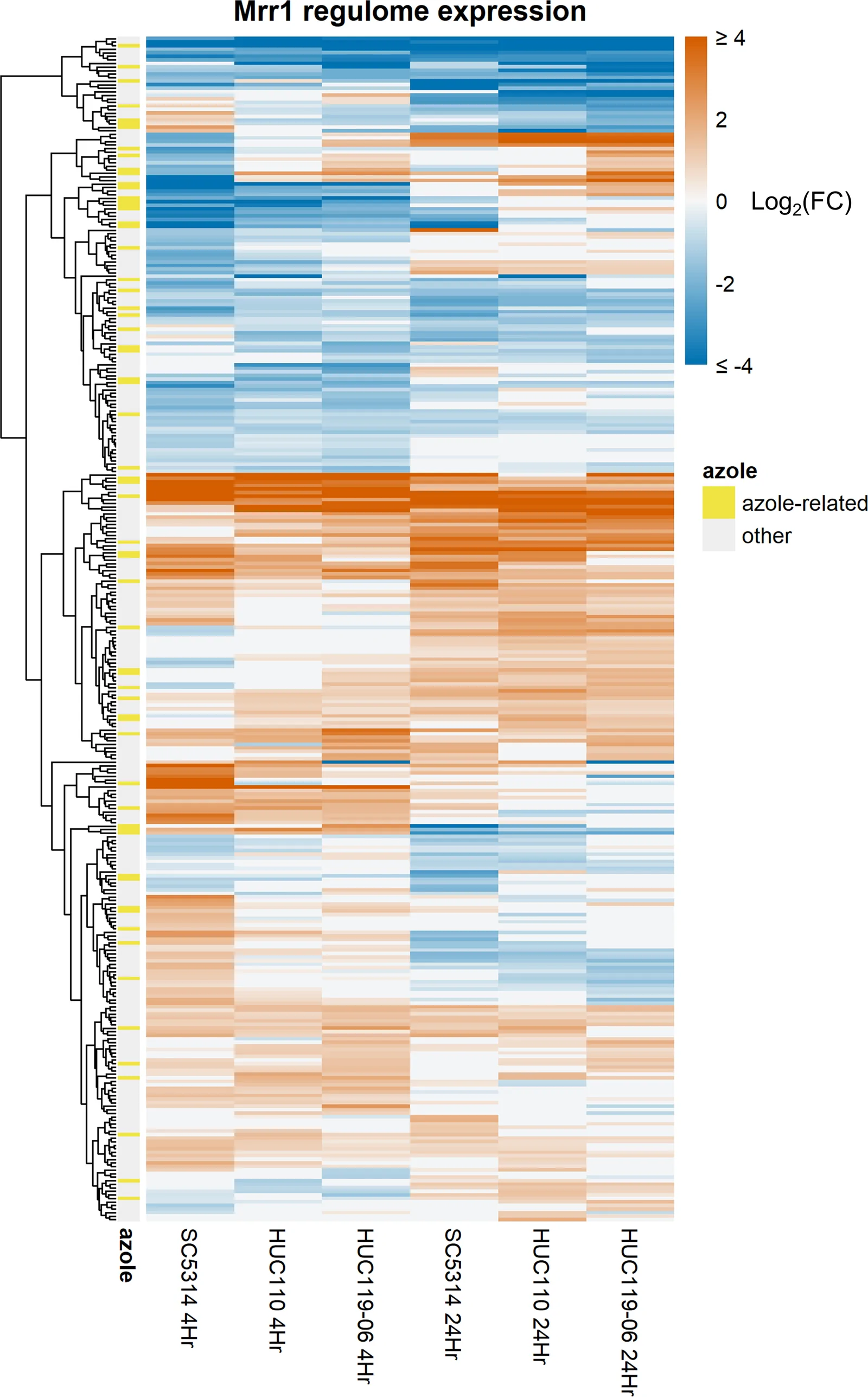
The Mrr1 regulon is enriched in AUM biofilms. Differentially expressed genes of SC5314, HUC110, and HUC119-06 grown in AUM biofilms vs RPMI biofilms were compared against the previously identified total regulon (all bound ORFs) of Mrr1 (72). Genes as being “azole-related” are those with the phrase “azole” used in the Assembly 22 annotation of the gene.

We compared the genes differentially expressed in AUM biofilms (Fig. 3) to the set of genes bound by Mrr1 using a hypergeometric test. There was significant enrichment for the Mrr1 regulon amongst the genes both upregulated and downregulated during growth in AUM for all three strains at both time points (Table 2; Fig. 5). One notable member of the Mrr1 regulon is *ALS1*, which was upregulated for all conditions except HUC119-06 at 24 h and is key for biofilm formation in murine CAUTI models (7, 34). *BRG1* and *EFG1*, members of the master biofilm regulatory network (73), are both directly regulated by Mrr1 (72), but only *BRG1* was differentially expressed in this data set. Many genes involved in azole resistance or differentially expressed due to exposure to azoles are regulated by Mrr1, and these are highlighted in Fig. 5. The enrichment of the Mrr1 regulon in genes differentially expressed in AUM biofilms is not direct evidence of Mrr1 activity but would be consistent with Mrr1 activating the transcription of genes to adapt to the urinary tract environment.

**TABLE 2:** Enrichment of the Mrr1 regulon in differentially expressed genes of AUM biofilms^*a*^

Sample	DE genes bound by Mrr1 (*n*)	Fold enrichment	*P* value
SC5314 4 h Up	110	1.4229	2.90E−05
SC5314 4 h Down	93	2.0273	2.58E−11
HUC110 4 h Up	74	1.6499	7.14E−06
HUC110 4 h Down	66	2.2480	3.42E−10
HUC119-06 4 h Up	94	1.8093	3.14E−09
HUC119-06 4 h Down	72	1.7599	1.09E−06
SC5314 24 h Up	94	2.0717	2.44E−12
SC5314 24 h Down	76	2.0815	3.42E−10
HUC110 24 h Up	98	2.0196	2.44E−12
HUC110 24 h Down	64	1.4252	0.0022
HUC119-06 24 h Up	99	2.0438	2.44E−12
HUC119-06 24 h Down	66	1.3390	0.0069

^*a*^ Enrichment was assessed using a one-sided hypergeometric test. Statistical significance and adjusted *P* values were calculated using the Benjamini-Hochberg false discovery rate test.

### Mrr1 provides a fitness advantage for growth in AUM for HUC110

To complement the genomic and transcriptomic data, we performed a competition assay between an *mrr1*Δ/Δ mutant and a WT strain. We selected HUC110 as an appropriate strain background for this experiment. The media tested were PHU, AUM, and an AUM formulation lacking the urea component (w/o urea), with or without 4 µg/mL of fluconazole, eight times the MIC for SC5314. We assayed the relative fitness via the ratio of tdTomato WT: *mrr1*Δ/Δ mutant determined by diluting and plating cells on YPD agar plates and then fluorescence and brightfield imaging followed by automated cell counting using ImageJ (representative images in Fig. S4A and B).

**Fig 6:**
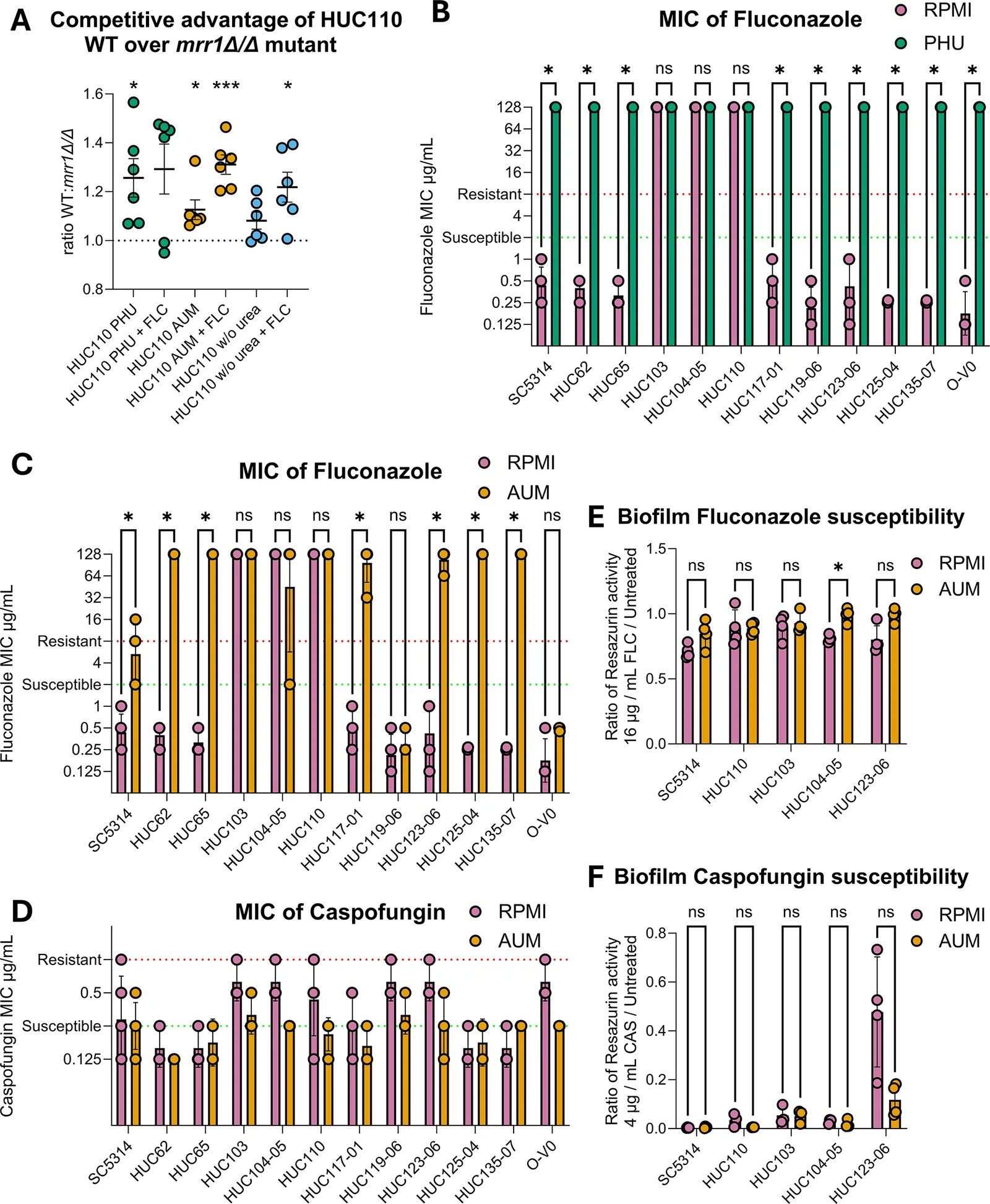
Growth in urine and artificial urine medium (AUM) increases *C. albicans* resistance to fluconazole. (**A**) Competition assays between WT HUC110 (tagged with tdTomato) and HUC110 *mrr1*Δ/Δ in urine, AUM, and an AUM formulation lacking urea ±4 µg/mL of fluconazole. Statistics represent *t*-test against a hypothetical data set of equal variance and ratio of WT:*mrr1*Δ/Δ of 1.0. *P < 0.05, **P < 0.01, and ***P < 0.001. (**B and C**) Planktonic MICs for the CA isolates were determined using the standard CLSI method in (**B**) RPMI vs urine and (**C**) RPMI vs AUM for fluconazole. The RPMI data in panels **B** and **C** are identical. (**D**) Planktonic MICs were determined in RPMI vs AUM for caspofungin. Planktonic MICs were determined as the amount of drug needed to reduce the OD_600_ by (**B and C**) 80% for fluconazole and (**D**) 90% for caspofungin. (**E and F**) Biofilm susceptibilities were determined for SC5314 and a set of CA isolates in RPMI vs AUM for (**E**) 16 µg/mL fluconazole and (**F**) 4 µg/mL caspofungin by treating 8-h biofilms with drug and growing for an additional 40 h. Relative biofilm viability was determined by measuring resazurin fluorescence. For panels **B–D**, differences between strains grown in RPMI and urine/AUM were determined using lognormal *t*-tests (Welch’s), and for panels **E and F** differences between strains grown in RPMI and AUM were determined using normal unpaired *t*-tests. For panels **B–F**, multiple comparisons were calculated using the Holm-Sidak method with an alpha value of 0.05; Adjusted *P* values: *P < 0.05. ns, not significant (*P* > 0.05).

If Mrr1-regulated efflux activity were important for adaptation to the high osmolarity of the urinary tract, we would expect that the *mrr1*Δ/Δ mutant would have a loss of fitness in the PHU and AUM environment but not in the AUM lacking urea, and this phenotype should be enhanced by the presence of fluconazole. In PHU, we measured WT to have a 25.6% competitive advantage against *mrr1*Δ/Δ. The AUM provided a 12.6% competitive advantage, and the addition of fluconazole increased this to 31.1%. The AUM without urea did not provide a competitive advantage for WT over the *mrr1*Δ/Δ mutant, but addition of fluconazole increased the competitive advantage to 21.9% (Fig. 6A). This experiment exhibited substantial variation between distinct lots of PHU, however; the trends for the PHU samples and AUM samples are similar. These data indicate that *MRR1-*regulated efflux plays a role in how HUC110 adapts to the high osmolarity environment of the urinary tract and this can be abrogated by removing urea.

### Growth in urine and artificial urine increases *C. albicans* resistance to fluconazole

Both the transcriptomic and genomic data suggest that the urinary environment may be associated with increased antifungal resistance. Thus, we performed antifungal susceptibility tests with fluconazole and caspofungin using our panel of 11 CA isolates. While most of the isolates came from patients with a recent history of antibacterial therapy, none had received antifungals in the last year (15, 16, 42). Using the standard CLSI microdilution minimum inhibitory concentration (MIC) assay (43), we found that the majority of the isolates are susceptible to treatment with fluconazole (Fig. 6B). Three strains, however, were completely resistant (HUC103, HUC104-5, and HUC110; Fig. 6B). This is more than one would expect from expectations based on population studies, but this is a small set of strains. The standard assay uses MOPS-buffered RPMI as the growth medium (43), and we compared this to MIC assays performed in PHU and AUM. When grown in PHU, all the strains were resistant, even at concentrations up to 128 µg/mL (Fig. 6B). Similarly, when grown in AUM, most, but not all, CA isolates demonstrated fluconazole resistance (Fig. 6C). Because this technique measures cells growing in a planktonic state, the phenotypes of biofilms that promote antifungal tolerance, including senescent cells in the population and the development of extracellular matrix (74–76), do not confound the results. Therefore, the environment-dependent fluconazole resistance phenotype observed here is consistent with a model that growth in urine media increases efflux processes as observed in our transcriptional data. Additionally, these results suggest that fluconazole is not the ideal choice for treatment of UTIs.

In contrast to fluconazole, caspofungin’s mechanism of action makes it less susceptible to efflux out of the cell (77). Therefore, we expected that if the regulation of efflux pumps is the primary mechanism by which these isolates achieve fluconazole resistance in urine, they would not have a change in caspofungin resistance. This was indeed the case as changing the media from RPMI to AUM caused no change in MIC, and all the isolates were susceptible to caspofungin in both conditions (Fig. 6D).

### Biofilms grown in artificial urine are resistant to fluconazole but not caspofungin

Biofilm growth is widely appreciated to confer increased antibiotic tolerance (74–76). To test this phenotype in urinary biofilms, we measured antifungal susceptibility using a resazurin viability assay. Consistent with previous reports (78, 79), our RPMI-grown SC5314 biofilms had only a minimal decrease in viability after fluconazole treatment, and this was also true in AUM (Fig. 6E). In addition to SC5314, we also tested HUC110 and three other CA strains that were strong biofilm formers in the *in vitro* catheter assay (Fig. 2A), HUC103, HUC104-05, and HUC123-06. All the clinical CA isolate biofilms were resistant to fluconazole in both conditions. These data, particularly the resistance of SC5314 and HUC123-06 AUM biofilms, are consistent with previous work measuring the effect of fluconazole in an *in vivo* murine CAUTI model that found only a minimal reduction in CFU of a nominally azole-sensitive strain after fluconazole treatment (34). In contrast to fluconazole, caspofungin virtually sterilized the biofilms with the exception of HUC123-06 grown in RPMI (Fig. 6F).

### The urinary environment can promote fluconazole resistance in other *Candida* species

We tested whether the environment-dependent fluconazole resistance observed in *C. albicans* was species specific or generalizable across pathogenic *Candida* species. Due to the wide range of species included in this analysis, we tested fluconazole MICs using both broth microdilution and Etest strips on solid media. Consistent with previous results, the Etest strips did not always result in the same MIC measurement (43, 80), but the trends were generally the same (Fig. S5A), adding rigor to our antifungal susceptibility testing.

**Fig 7:**
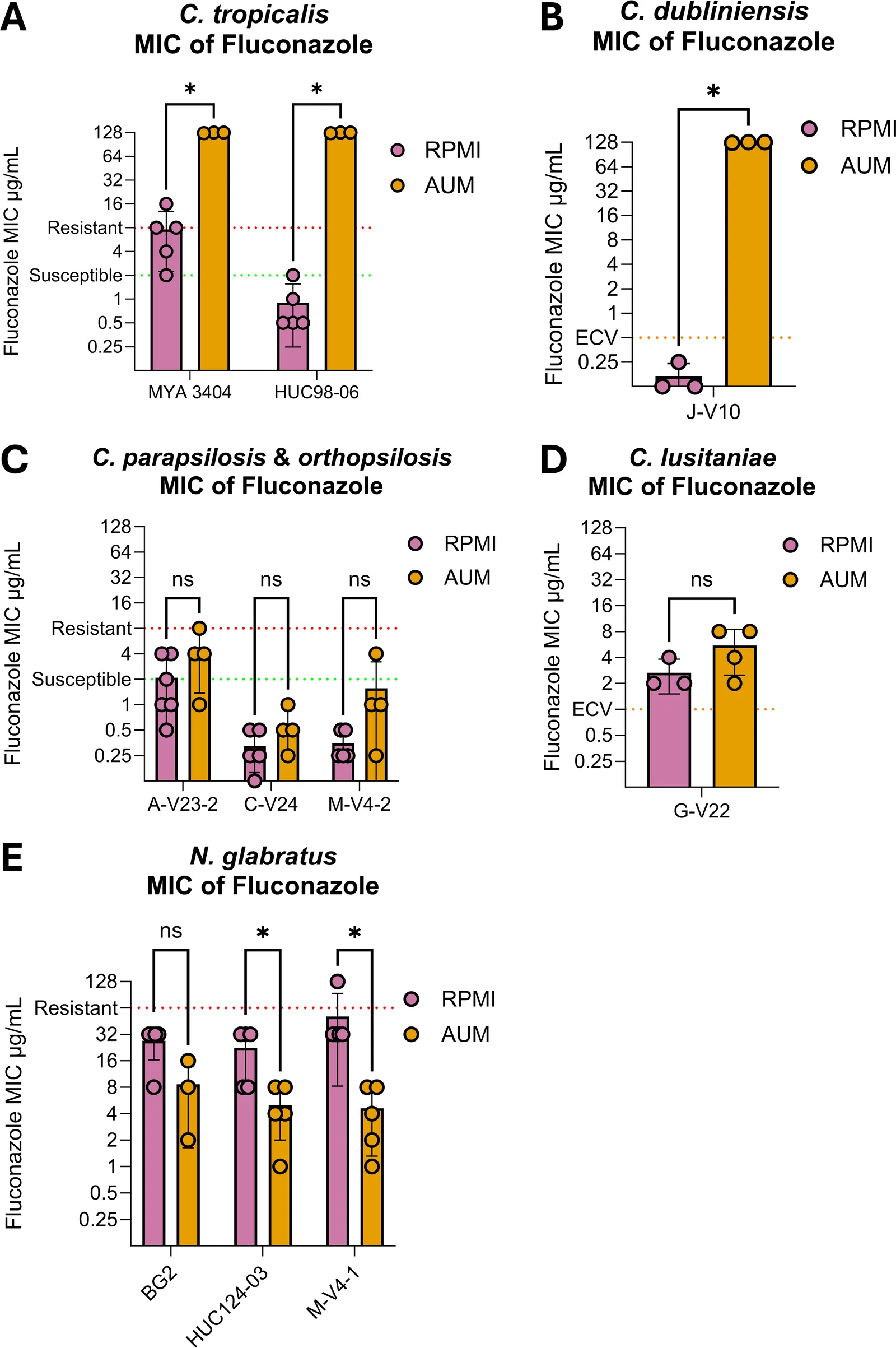
The urinary environment promotes fluconazole resistance in some, but not all, *Candida* species. (**A–E**) Planktonic MICs for the non-*albicans* catheter-associated isolates were determined using the standard CLSI method in RPMI vs AUM for fluconazole. Planktonic MICs were determined as the amount of drug needed to reduce the OD_600_ by (**A–D**) 80% for all species except (**E**) *N. glabratus,* which was set to 50% inhibition. For panels **A–E**, differences between strains grown in RPMI and AUM were determined using lognormal *t*-tests (Welch’s), and multiple comparisons were calculated using the Holm-Sidak method with an alpha value of 0.05; Adjusted *P* values: *P < 0.05. ns, not significant (*P* > 0.05). MIC values were compared to established clinical breakpoints (81) or epidemiological cutoff values (82) for species lacking rigorous clinical breakpoints.

*Candida tropicalis* and *Candida dubliniensis* are closely related to *C. albicans,* and both demonstrated increased fluconazole resistance in AUM compared to RPMI. In RPMI, the *C. tropicalis* laboratory strain MYA-3404 had an MIC of 8 µg/mL, which is at the clinical breakpoint for resistance (81), and the CA clinical isolate HUC98-06 had an MIC of 0.5 µg/mL. Both strains have significantly higher MICs of >128 µg/mL in AUM (Fig. 7A) but were susceptible to caspofungin in this medium (Fig. S5B). The *C. dubliniensis* CA isolate J-V10 grown in RPMI displayed an MIC of 0.12 µg/mL, but its MIC was significantly higher in AUM (128 µg/mL) (Fig. 7B). There was no change in the caspofungin MIC for J-V10 between media (Fig. S5C).

More distantly related from *C. albicans*, members of the *Candida parapsilosis* species complex isolated from urinary catheters (*C. orthopsilosis* A-V23-2; *C. parapsilosis* C-V24 and M-V4-2) were all susceptible to fluconazole (81) in RPMI (MICs of 2, 0.25, and 0.25 µg/mL, respectively) and showed no difference in fluconazole resistance in AUM by broth microdilution (Fig. 7C). These CA isolates show significantly higher MICs on solid AUM compared to RPMI by Etest (Fig. S5A). These species exhibit intrinsically higher baseline tolerance to echinocandin treatment due to a polymorphism in *FKS1* (83). All three CA isolates demonstrated paradoxical growth (growth rebound at high caspofungin concentrations, also known as the “Eagle effect”) in RPMI, which has been observed previously in the literature (84, 85). However, these strains did not have this phenotype in AUM, where the MICs were also lower (Fig. S5C). AUM is only the second medium reported to eliminate the paradoxical growth phenotype, with the other being the addition of human serum (86).

A single *C. lusitaniae* CA isolate, G-V22, displayed MICs above the epidemiological cutoff value (ECV) (82) in both RPMI (2 µg/mL) and AUM (4 µg/mL), but this difference was not statistically significant (Fig. 7D). Fluconazole MICs measured by Etest did show a significant increase when grown on AUM (Fig. S5A). G-V22 displayed caspofungin MICs above the ECV (82) in both RPMI and AUM with no significant difference between the two media (Fig. S5D).

*Nakaseomyces glabratus*, formerly *Candida glabrata*, is the second most common fungal pathogen implicated in CAUTIs (18). Of the CA isolates collected, it is the most distantly related to *C. albicans* and is not a member of the CUG clade, unlike all the previously discussed fungal pathogens. It has an intrinsically higher threshold for efficacy for fluconazole, and it can utilize unique mechanisms to achieve resistance, such as exogenous sterol uptake (87). We tested two CA isolates, HUC124-03 and M-V4-1, for fluconazole susceptibility along with the historical laboratory strain BG2. All three strains had MICs of 32 µg/mL in RPMI (Fig. 7E), which is the susceptible–dose-dependent cutoff (81). The *N. glabratus* strains only displayed minimal growth in the AUM. Because the growth was so low, we lowered our MIC calling threshold to 50% inhibition in AUM, and the two CA isolates had significantly lower MICs in AUM (Fig. 7E). Caspofungin susceptibility remained unchanged based on MICs between the two media, with all three strains displaying resistance (Fig. S5E).

These data demonstrate that environment-dependent fluconazole resistance is not unique to *C. albicans* but can be found in other species of the CUG clade; however, this trait was most strongly observed in species closely related to *C. albicans*. These data suggest that several CUG clade species utilize efflux pump activity to adapt to osmotic stress in the urinary tract.

## DISCUSSION

CAUTIs are the most common nosocomial infection in developed countries, and some reports indicate *Candida* species are the most commonly isolated organisms from CAUTIs, with *C. albicans* by far the most frequent (1, 18, 19). Despite this, virtually nothing is known about the molecular mechanisms of virulence, adhesion, drug resistance, or polymicrobial interactions in the urinary environment. To begin to address these knowledge gaps, we created a novel AUM to improve reproducibility between experiments relative to pooled or standardized human urine. This medium is made distinct from the formulation that inspired it (41) by the inclusion of all the micronutrients necessary for *Candida* growth in physiologically relevant concentrations compared to healthy human urine. We show here that this AUM is comparable in most parameters to PHU, while being more reproducible. While this medium was designed primarily with an interest in *Candida* cultivation, it also supports the growth of gram-negative and gram-positive pathogens (Fig. S1A and B), demonstrating its broad applicability.

We pair this novel AUM with several CA *C. albicans* isolates from two separate collections (15, 16, 42). There is some variability in phenotypes in these isolates, notably in biofilm formation on silicone catheters in AUM, in which clusters of high- and lower-biofilm formers were observed. The reference strain SC5314 was among the high biofilm formers and was otherwise similar to the clinical isolates in most assays, suggesting that it is an appropriate strain for CAUTI-related studies. Our transcriptomic studies support this conclusion: there was remarkable similarity in the transcriptomes of mature (24-h) biofilms between SC5314 and two CA strains with disparate biofilm phenotypes across various media and substrate conditions. In contrast, the transcriptional differences between AUM and RPMI were considerable, highlighting the niche-specific adaptation in this developmental program, as has been previously observed (73, 88, 89). In AUM, GO analysis revealed that processes involved in xenobiotic detoxification were consistently upregulated in all three strains.

The overrepresentation of “xenobiotic detoxification” and efflux pump genes, along with polymorphisms in known regulators of drug efflux, led us to ask whether the urinary environment would alter antifungal susceptibility relative to other media conditions. Indeed, it does: all our CA isolates met clinical breakpoints for fluconazole resistance in PHU, while only a subset were resistant in RPMI. None of the individuals from whom these strains were collected had received antifungal therapy for at least one year prior to collection (15, 16), suggesting the resistance phenotype in urine arose from other stimuli. This resistance phenotype was also seen in other fungal species isolated from long-term urinary catheters, more commonly in species closely related to *C. albicans*. However, we had access to a limited number of non-*albicans* CA strains. Further research with a larger collection of clinical isolates would refine these conclusions. Together, these results suggest that the urinary environment may select for azole resistance and, regardless of laboratory susceptibility testing, fluconazole may not be the optimal therapeutic option for *Candida* CAUTI.

Whole-genome sequencing of the CA clinical isolates revealed variant alleles of *MRR1* that have been previously correlated with azole resistance in all but one of these strains (44, 65–67). Mrr1 is a key transcriptional regulator of antifungal resistance via regulation of MDR efflux pumps. Previous studies have examined the selective pressure for *MRR1* gain-of-function in *C. lusitaniae* in the antifungal-naïve cystic fibrosis lung environment, where they correlated *MRR1* sequence variants with fluconazole susceptibility (45, 71). In this system, the selection for gain-of-function *MRR1* alleles was attributed to exposure to the toxic glycolytic byproduct methylglyoxyl. Here, we present evidence that Mrr1 regulates detoxification or efflux mechanisms that are necessary for growth in the high salt, high osmolarity urinary environment, and propose that urea is a driving force. Thus, the transcriptional regulator *MRR1* appears to be critical for creating the necessary transcriptional program for these isolates to grow in the urinary environment. We found it interesting that all but two of the CA clinical isolates are heterozygous for the *MRR1* locus. Previous work has shown that fluconazole treatment promotes gain-of-function mutations in one allele followed by loss of heterozygosity (44). The heterozygosity we observe is consistent with an intermediate step in this process and suggests a substantial pressure on the *MRR1* locus in the urinary tract environment. It is notable that polymorphisms in other azole-resistance regulators, such as *MRR2*, *UPC2*, and *TAC1*—but not in the azole target *ERG11*—were also identified, suggesting that selection pressures for efflux activity specifically are considerable. The summation of the data presented here supports a model in which *C. albicans* increases efflux activity to adapt to the environmental stresses of the urinary tract, such as high osmolarity and urea concentrations, and a corollary benefit of this activity is an increased resistance to fluconazole.

Several of the CA clinical isolates underperformed in the *in vitro* urinary catheter biofilm assay, while none were deficient in biofilm production in other media. This suggests that the *in vitro* conditions do not fully recapitulate the CAUTI environment. Unique host factors and, critically, the unique microbiota present in each catheterized bladder may affect the biofilm developmental program in positive or negative ways. Importantly, all of the *C. albicans* CA strains were co-isolated with one or more bacterial strains, which may impact morphogenesis, adhesion, biofilm formation, and drug resistance. In contrast, the upregulation of efflux and increased antifungal resistance was consistent for the majority of the CA isolates tested, indicating this phenotype is more strongly under selective pressure in the urinary tract and perhaps independent. Additional studies are critically needed to dissect these mechanisms.

Notably, HUC119-06 did not form strong biofilms *in vitro* on silicone urinary catheters (Fig. 2A), did not have a change in fluconazole MIC when grown in AUM (Fig. 6C), nor did it persist in the urinary tract of the individual from whom it was isolated (15). The early STOP codon in one of its alleles of *MRR1* may provide an explanation for these phenomena. Further work is needed to determine if this gene dosage effect is responsible for the lower biofilm formation and fluconazole susceptibility in HUC119-06.

In summary, the urinary environment is a unique and understudied niche with significant clinical implications, most notably in its implications in antifungal chemotherapy. The biofilm transcriptional program in urine differs significantly from RPMI, or other conditions that are better understood. We present here a new synthetic media (AUM) to standardize conditions between labs and use it to demonstrate that the lab strain SC5314 is a reasonable model for CAUTI studies. Finally, we identify that urine promotes azole resistance and that this is due, in part, to selection for mutations in the transcriptional regulator Mrr1, as has been observed in other models (44, 65–67). Future studies—including in murine CAUTI models—will dissect these selection mechanisms and explore other unique emergent properties of *Candida* biofilm growth in the catheterized urinary tract to better understand and treat these infections.

## MATERIALS AND METHODS

### Strains

The identification numbers, genotypic information, and origins of strains used in this study are listed in Table S1 in the Supplemental Material. CA clinical isolates were a gift from S. Hultgren (15, 42) and C. Armbruster (16). The naming convention for these strains when used in the literature refers to the participant number and time point at which these strains were collected.

Fungal colonies were maintained at 25°C on YPD (1% yeast extract, 2% peptone, and 2% dextrose) agar plates and grown in liquid YPD at 30°C with aeration for at least 18 h to stationary phase prior to use in experiments (90). Bacterial colonies were maintained at 4°C on Luria broth (LB) (91) or brain heart infusion (BHI) (92) agar plates and grown in liquid media of the same type at 37°C with aeration for at least 18 h to stationary phase prior to use in experiments.

### Mutant generation

Deletion mutants were created as previously described using CRISPR-Cas9-directed mutagenesis and a flippable nourseothricin resistance cassette, thus leaving an empty locus and a strain background that is both prototrophic and nourseothricin sensitive (93–95).

### Human urine collection

Human urine was sourced from the Urine BioBank for Urinary Tract Infection Studies, in accordance with protocol IRB #HSC-MS-21-0849 approved by the CPHS at the University of Texas Health Science Center at Houston (UTHealth). Briefly, donors consented and were enrolled prior to donation. Urine from at least two each of female and male donors, aged 18+ were collected and pooled. Donors were not taking antimicrobials, nor currently menstruating, pregnant, nor experiencing symptoms of a UTI. Urine was sterilized with a 0.22 µm filter (Thermo) and stored at 4°C for use within 72 hours. Excess urine was frozen at −80°C for long-term storage and thawed for immediate use.

### Media and buffers

Our novel AUM was made using the base formula described in references 41, 41 with additions as described in Table 1. Physiologically relevant amounts of glucose (96–98), biotin (99), tryptone (to account for the absent proteins) (96, 100), and yeast nitrogen base (to account for absent vitamins and minerals excreted in urine) (96) were added to the base formula. Each ingredient of the AUM was added as a dry component with the exception of biotin and potassium oxalate, which were kept as separate stock solutions. The medium was sterilized by vacuum filtration and stored at room temperature away from light for up to 3 months.

The Brooks artificial urine medium was prepared as previously described (38). The AU-Siriraj medium was prepared as previously described (39). RPMI-1640 with l-glutamine without sodium bicarbonate (Corning #50-020-PC) was used for biofilm assays. For planktonic MIC assays, RPMI was buffered with MOPS [3-(N-morpholino) propanesulfonic acid] at a concentration of 0.165 mol/L, and the pH was adjusted to 7.0 (43). Spider medium was made as has been previously described (101). YNB 2% glucose was made by combining 2% (wt/vol dextrose, 5 g (NH_4_)_2_SO_4_, and 1.7 g yeast nitrogen base (Difco #233520) to 1 L H_2_O and sterilizing by filtration. Phosphate-buffered saline (PBS) without calcium and magnesium was used (Corning #21-040-CV).

### Growth curves

Stationary phase cultures were subcultured for 4–6 h in YPD, LB, or BHI. Cells were pelleted by centrifugation for 2 min at 5,500 rcf and washed twice with PBS. These cells were diluted in PBS to a standard starting concentration of approximately 0.04 as measured by optical density at 600 nm (OD_600_) of 200 µL of liquid in a clear flat-bottom 96-well suspension culture plate (Falcon). Then, 10 µL of these standard starting solutions was added to 190 µL of the final culturing media in 96-well suspension culture plates. Plates were sealed with Breathe Easy membranes and incubated in a Synergy H1 plate reader (Bio-Tek, USA) at 37°C with continuous shaking, and the OD_600_ was measured every 15 or 20 min for 24 h. These experiments were performed with four technical replicates and at least three biological replicates. Area under the curve was calculated using GraphPad Prism 10.0; statistical analysis was performed using a one-way analysis of variance (ANOVA) test and a Sidak’s multiple comparisons test to compare groups (GraphPad Prism 10.0).

### Ninety-six-well plate biofilm assays

For biofilms grown in PHU or AUM (but not RPMI or Spider), the wells of 96-well tissue culture treated polystyrene plates were pre-coated with human Fg (Sigma) as described previously (48). The Fg was diluted to 100 µg/mL into warm 50 mmol/L NaHCO_3_ and allowed to adhere to the plate for at least 18 hours at 4°C before removing prior to the start of the experiment. Biofilm biomass quantification was performed using a standard method (102). The day following Fg coating (if needed), stationary phase cultures were subcultured for 4–6 h in YPD. Cells were pelleted by centrifugation for 2 min at 5,500 rcf and washed twice with PBS. These cells were diluted in PBS to a cell density of approximately 5 × 10^6^ cells/mL (measured with Countess II, Life Technologies). Then, 100 µL of the cell suspension was added to wells of a 96-well tissue culture treated polystyrene plate (Falcon). This was incubated for 1 h at 37°C with orbital shaking at 200 rpm. The PBS was gently removed and replaced with 200 µL of test culture media. For PHU and AUM biofilms, the medium was supplemented with 100 µg/mL Fg. Plates were sealed with a Breathe Easy membrane and incubated statically for 48 h at 37°C. The medium was removed and biofilms were dried at 37°C for an hour. Biofilms were then stained with 75 µL of 0.1% crystal violet (diluted, Sigma) for 30 min. Crystal violet was removed and biofilms were washed at least twice by gentle additions of water. Biofilms were destained with 200 µL of 95% ethanol for 20 min. Then, 75 µL of this destain was transferred to clean wells of a new plate and diluted with addition of 125 µL of water. Final quantification was done by measuring the absorbance at 595 nm (OD_595_) using a Synergy H1 plate reader (Bio-Tek). Experiments were performed with six technical replicates and at least three biological replicates. Staining of medium only control wells was subtracted as background and then absorbance measurements were converted to percentages relative to SC5314 for consistent comparison across biological replicates. Statistical analysis was performed in GraphPad Prism 10.0 using one-way ANOVA followed by Dunnett’s multiple comparison test. Lines with error bars indicate the mean and the standard deviation (SD).

### *In vitro* urinary catheter biofilm assay

Biofilm growth on human urinary catheters was measured by optimizing an established protocol (48) for use with *Candida albicans*. The day prior to the start of the experiment, size 16 Fr Foley catheters (McKesson MFR #16-SF0L516) were cut into 2 cm sections and then bisected along the long axis to create two half cylinder sections. Catheter sections were soaked in a 100 µg/mL Fg (Sigma) and 50 mmol/L NaHCO_3_ solution for at least 18 h at 4°C. The following day, catheter pieces were transferred to individual glass tubes, each with 5 mL of AUM and 100 µg/mL of Fg. On the same day, stationary phase cultures were subcultured for 4–6 h in YPD at 30°C with aeration. Cells were pelleted by centrifugation for 2 min at 5,500 rcf and washed twice with PBS. These cells were diluted in PBS to a cell density of approximately 5 × 10^6^ cells/mL (measured with Countess II, Life Technologies). Catheter tubes were inoculated with 50 µL of the cell suspension. Samples were incubated for 48 h at 37°C with orbital shaking at 200 rpm in a standing tube rack. After incubation, catheters were gently removed from medium and allowed to dry for 24 h before being transferred to 1.5 mL tubes and stained with 0.1% crystal violet (diluted, Sigma) for 20 min. Excess dye was washed away by submerging catheters in sterile water and dabbing with a paper towel. Catheters were transferred to sterile tubes and destained with 70% ethanol/30% acetic acid. Then, 100 µL of this destain was transferred to clean wells of a new plate and diluted with addition of 100 µL of water. Final quantification was done by measuring the absorbance at 595 nm (OD_595_). Experiments were performed with three technical replicates and at least three biological replicates. Staining of medium only control wells was subtracted as background and then absorbance measurements were converted to percentages relative to SC5314 for consistent comparison across biological replicates. Statistical analysis was performed in GraphPad Prism 10.0 using one-way ANOVA followed by Dunnett’s multiple comparison test. Lines with error bars indicate the mean and the standard deviation (SD).

### Transcriptome analysis

The preparation of biofilms, extraction and isolation of RNA, and analysis were performed using a similar method as our previously published protocol (103) with the following changes for use on biofilms grown in AUM and RPMI. The day prior to the start of the experiment, tissue culture (TC) flasks (surface area 25 cm^2^) (Thermo Scientific #156367) were coated with 7.8 mL of 100 µg/mL Fg (Sigma) in 50 mmol/L NaHCO_3_ solution for at least 18 h at 4°C. The following day stationary phase cultures were subcultured for 4 h in YNB 2% glucose at 30°C with aeration. Cells were pelleted by centrifugation for 2 min at 5,500 rcf and washed twice with PBS. These cells were diluted in PBS to a cell density of approximately 5 × 10^6^ cells/mL (measured with Countess II, Life Technologies). The Fg coating solution was removed from the flasks and replaced with 8 mL of the PBS cell suspension. This was incubated for 1 h at 37°C with orbital shaking at 120 rpm. The PBS was gently removed and replaced with 15 mL of warmed media (either RPMI or AUM). Biofilms were grown statically for either 4 or 24 h before being scraped off the TC flask surface and washed once with PBS. RNA isolation was performed using the RiboPure Yeast RNA Extraction Kit (Ambion). Sample quality and concentration were assessed using the Qubit RNA IQ (Invitrogen) and Qubit RNA High Sensitivity Assay (Invitrogen) kits. The mRNA was sequenced by the UTHealth Houston Cancer Genomics Center (CPRIT RP240610) using paired-end reverse-strand Illumina short-read sequencing.

Data analysis was performed using the North American Galaxy server using a well-established transcriptome analysis pipeline (104–106). Adapter sequences were trimmed using Trim Galore! 0.6.10 + galaxy0. Sequencing reads were aligned to a haploid Assembly 22 genome sequence of SC5314 using RNA STAR v2.7.11b + galaxy0. Gene expression was counted using featureCounts v2.1.1 + galaxy0, and differential expression analysis was performed using DESeq2 v2.11.40.8 + galaxy0. Differentially expressed genes with a log_2_(fold change) >2 or <0.5 and adjusted *P* value <0.01 were considered significant. GO analysis was performed on the Candida Genome Database server (107). Downstream analyses and data visualization were performed using R.

### Genomic analysis

Whole-genome sequencing was performed by SeqCenter using paired-end Illumina short-read sequencing. Adapter sequences were trimmed using Trim Galore! 0.6.10 + galaxy0. Trimmed reads were mapped to a haploid sequence of SC5314 Assembly 22 using BWA-MEM 0.7.17-r1188 (108).

### Population genomics analysis

Placement of the CA isolates within the *C. albicans* population structure was determined as has been previously described (62, 109). Briefly, whole-genome sequencing reads were coordinate-sorted and indexed with samtools (v1.19.2). Duplicates were flagged using GATK (v4.6.2.0) with Picard (v3.4.0) MarkDuplicates. Variants were called using GATK HaplotypeCaller (v4.6.2.0) in GVCF mode with the sample ploidy set to two. Individual GVCFs were combined with CombineGVCFs and jointly genotyped across all samples using GenotypeGVCFs. Raw SNPs were extracted with GATK SelectVariants and subjected to hard filtering using GATK VariantFiltration. Sites were excluded if they met any of the following criteria: QualByDepth (QD) < 2.0, FisherStrand (FS) > 60.0, RMS mapping quality (MQ) < 40.0, MQRankSum < −12.5, or ReadPosRankSum < −8.0. Per-genotype filters were then applied using bcftools (v.1.19) (110). Individual genotype calls with a genotype quality (GQ) below 20 or read depth (RD) below 10 were set to “missing.” Sites with a missing genotype rate exceeding 10% across all samples were excluded from the final call set.

Publicly available whole-genome sequencing data were obtained from the European Nucleotide Archive (ENA). A total of 40 isolates representing all major *C. albicans* clades (or 37 + 3 *Candida africana* isolates) were selected from reference 62 (BioProject PRJNA432884) with three representatives for most clades. These reads were trimmed with fastp (v.0.23.4) and then processed through the same pipeline as the CA isolates. All GVCFs from both the clinical isolates and the public data set were combined and jointly genotyped as a single cohort. The same filtering parameters as before were applied to the merged call set. The final data set contained 356,389 biallelic SNPs across 53 samples.

A concatenated SNP alignment was generated from the filtered VCF by converting each genotype to its corresponding nucleotide. Heterozygous positions were encoded using IUPAC ambiguity codes to preserve diploid genotype information. A maximum likelihood phylogeny was inferred using IQ-TREE (iqtree2 v2.0.7+dsfg-1) with the GTR + G4 substitution model with 1,000 ultrafast bootstrap replicates. The sequence type was specified as DNA to prevent misidentification of the SNP-only alignment. Ascertainment bias correction was not applied because a small number of invariant sites (*n* = 71) remained in the alignment after filtering. The tree was midpoint-rooted in R (v.4.5.2) using the phangorn package and visualized with ggtree.

### Gene variant analysis

Variants were called using bcftools 1.19 mpileup and call functions (110) with a minimum mapping quality of 20 and a minimum base quality of 20. Variants were filtered to retain only those with coverage ≥ 10× and quality scores ≥30. Variants were classified as homozygous with allele frequency ≥ 0.9, or heterozygous with allele frequency between 0.4 and 0.6. Aneuploidy was assessed by examining genome-wide coverage patterns normalized to the median coverage. Known antifungal resistance genes were extracted from the *C. albicans* genome annotation, and variants within these loci were translated to protein coordinates using custom R scripts. Sequence analysis and translation were performed using Biostrings 2.78.0 (111). Sequences were aligned using ClustalW 2.1 + lgpl-7 (112) and compared to known alleles associated with antifungal resistance in the literature.

### Planktonic broth microdilution MIC assays

The broth microdilution assay follows the protocol established by the Clinical and Laboratory Standards Institute (CLSI) (43) with a slight modification in the final cell concentration. Briefly, drug dilutions were performed by 1:1 serial dilution from stocks set at 128 and 8 µg/mL for fluconazole (Sigma-Aldrich) and 16 and 0.5 µg/mL for caspofungin acetate (Biosynth). After dilution, 190 µL of medium was added to 96-well U-bottom plates (Falcon) for fluconazole and 96-well flat bottom suspension plates (Falcon) for Caspofungin. Stationary phase cultures were pelleted by centrifugation for 2 min at 5,500 rcf and washed twice with PBS. These cells were diluted in PBS to a standard starting concentration of approximately 0.02 as measured by optical density at 600 nm (OD_600_) of 200 µL of liquid in a clear flat-bottom 96-well suspension culture plate (Falcon). The cell suspension was diluted by 1:10 in the relevant medium (RPMI-MOPS, AUM, or PHU) and then finally diluted 1:20 by adding 10 µL to each well containing 190 µL of the drug dilution medium. The plates were sealed with a Breathe Easy membrane and incubated statically for 48 h at 37°C. After incubation, the absorbance at 600 nm was measured with a Synergy H1 plate reader (Bio-Tek). For the U-bottom plates, nine OD measurements were taken from each well, and the mean measurement was used as the final OD_600_. Fluconazole MICs were determined to be the amount of drug that reduced the optical density by at least 80% compared to the untreated well. For the *Nakaseomyces glabratus* samples, this threshold was lowered to 50% in the AUM to account for the limited growth of this organism. Caspofungin MICs were determined to be the amount of drug that reduced the optical density by at least 90% compared to the untreated well. Experiments were conducted with at least three biological replicates. Statistical analysis for each strain in different media was performed using lognormal *t*-tests with Welch’s correction, and multiple comparisons were calculated using the Holm-Sidak method with an alpha value of 0.05 (GraphPad Prism 10.0). MIC values were compared to established clinical breakpoints (81) or epidemiological cutoff values (82) for species lacking rigorous clinical breakpoints.

### Biofilm antifungal susceptibility assay

To measure the efficiency with which antifungals kill fungal biofilms, we used a resazurin viability assay as previously described (113) with some optimizations as previously described (114) and some uniquely described here. Briefly, biofilms were prepared and incubated using mostly the same method as described here in the 96-well biofilm assay with the following modifications. We used black opaque polystyrene tissue culture treated 96-well plates instead of transparent plates, and we performed four technical replicates of untreated biofilms and four technical replicates for biofilms treated with either 16 µg/mL of fluconazole or 4 µg/mL of caspofungin. Biofilms were grown statically for 8 h before drug treatment, and an additional 40 h after drug treatment. After incubation, the medium was removed and replaced with freshly prepared, warmed, and sterilized PBS + 1% glucose + 0.003 g resazurin. Resazurin fluorescence was measured with an excitation of 550 nm and emission of 590 nm using a Synergy H1 plate reader (Bio-Tek). The plate was incubated at 37°C with shaking and measured every 15 min for 3 h. The measurement runtime was optimized for each strain as being the highest average fluorescence for the untreated samples. Different strains metabolize and eventually break down the resazurin dye at different rates, so it was necessary to optimize measurement timing for each strain tested, and as a consequence, it is not recommended that this technique be used to compare different strains (114, 115). Viability of drug-treated biofilms was determined by subtracting the background fluorescence of untreated wells and then taking the ratio of the mean relative fluorescence of the treated wells to the untreated wells. The experiment was conducted in at least biological triplicate. Statistical analysis was performed in GraphPad Prism 10.0 using unpaired *t*-tests. Lines with error bars indicate the mean and the standard deviation (SD).

### Competition assay

The HUC110 *mrr1*Δ/Δ mutant and a HUC110 WT strain constitutively expressing tdTomato were each grown following standard culturing and subculturing. Cells were washed, diluted, and then grown in pure cultures of the relevant test media (PHU, AUM, or AUM without urea) for 24 h at 37°C with aeration. Then samples were collected, and cells were counted (Countess II, Life Technologies). WT and mutant samples from each test medium were mixed in a 1:1 ratio by cell count, and then 100 µL of this mixture was added to fresh 5 mL cultures of each test medium. Fluconazole was added to relevant cultures at a concentration of 4 µg/mL. A culture of YNB 2% glucose was used as a non-restrictive control. The competition cultures were grown at 37°C for 48 h with aeration. Cells were again counted and diluted onto YPD plates. Plates were incubated for 48 h at 30°C. A gel imaging station (Bio-Rad, ChemiDoc) was used to take photographs of the plates in a fluorescent channel (rhodamine), followed by a bright exposure (Coomassie blue). This allowed us to image fluorescent WT colonies only, followed by total colonies. Colony counting was automated in ImageJ.

The experiment was performed with six biological replicates, each comprised of three technical replicates. The ratio of WT:*mrr1*Δ/Δ was calculated, and we created synthetic data to fit the null hypothesis of a 1:1 ratio of WT:*mrr1*Δ/Δ with the same variance of the real data. We performed Shapiro-Wilk tests for each experimental condition to assess the normality of the distribution of the WT:*mrr1*Δ/Δ ratio. For normally distributed groups, we used *t*-tests to assess individual comparisons of the WT:*mrr1*Δ/Δ ratio against the hypothetical neutral competition, and for non-normal groups, we used Wilcoxon tests. We used a two-way ANOVA to examine the interaction between fluconazole and media across all groups. The ANOVA confirmed that the addition of drug had a uniform effect across all three media.

### Etest MIC assay

Fluconazole susceptibility was measured with Etest strips following the standard CLSI method (43). Stationary phase cultures grown in YPD for at least 18 h were pelleted by centrifugation for 2 min at 5,500 rcf and washed twice with PBS. These were diluted in PBS to an OD_600_ of approximately 0.05, as measured by 200 µL of culture in a 96-well plate using a Synergy H1 plate reader (Bio-Tek). This cell suspension was streaked onto AUM and RPMI-MOPS plates with a sterile cotton swab, two strains to a plate, and an Etest strip (Liofilchem) was placed between the two swabbed areas. MICs were determined as the intersection between the zone of inhibition and the reading on the Etest strip, and the experiment was conducted with three biological replicates. Statistical analysis for each strain in different media was performed using lognormal *t*-tests with Welch’s correction, and multiple comparisons were calculated using the Holm-Sidak method with an alpha value of 0.05 (GraphPad Prism 10.0).

### Comparison of the Mrr1 regulon and transcriptomics data

The Mrr1 regulon was defined as the 717 binding sites identified previously by ChIP-chip (72). Binding sites with no assignable open reading frame (ORF), located ambiguously between reading frames, or assigned to reading frames that have been recategorized in Assembly 22 (i.e., combined with another ORF, removed) were removed from the list, leaving 454 unique Mrr1-bound ORFs. The Mrr1 regulon was compared against the DESeq2 output files from the SC5314, HUC110, and HUC119-06 AUM biofilm mRNA sequencing experiment. Genes with NA adjusted *P* values (removed by DESeq2 filters for low counts) were excluded from all downstream tests. Among tested genes, those with log_2_FC ≥1 and adjusted *P* <0.01 are classified as upregulated DEGs, and those with log_2_FC ≤−1 and adjusted *P* <0.01 as downregulated DEGs, and the union as DEGs in either direction. Statistical comparison between the Mrr1 regulon and AUM biofilm DEGs was performed using a hypergeometric enrichment test. For each comparison, three one-sided hypergeometric tests were performed using the R (v.4.5.2) function “phyper(…, lower.tail = FALSE).” The fold enrichment value was calculated only using counts of DEGs, not mean expression magnitude. *P* values were adjusted using the Benjamini-Hochberg false discovery rate procedure applied independently within each direction (up, down, and either). The composite heatmap was generated using the R package pheatmap v1.0.13. Genes identified as “azole-related” are those with the phrase “azole” in their description in the GFF gene annotation file for Assembly 22 (107).

## Data Availability

The raw transcriptional and genome sequence data have been uploaded to the Sequence Read Archive at NCBI with project accession number PRJNA1491355.

## SUPPLEMENTAL MATERIAL

Supplemental Material mbio.01785-26-s0001.pdf Table S1 and Figures S1-S5.

Table S2 mbio.01785-26-s0002.xlsx Transcriptomic data.

ASM does not own the copyrights to Supplemental Material that may be linked to, or accessed through, an article. The authors have granted ASM a non-exclusive, world-wide license to publish the Supplemental Material files. Please contact the corresponding author directly for reuse.
