# Supplementary material for: The catheterized urinary tract selects for MRR1-mediated efflux and fluconazole resistance in Candida albicans biofilms
Source: mBio. 2026 Sep 14;17(10):e01785-26. doi: 10.1128/mbio.01785-26 (PMC13712843; doi:10.1128/mbio.01785-26)
Supplement: Supplementary file 1 [file mbio.01785-26-s0001.pdf]

Table S1. Strains used in this study

Strain	Species	Relevant genotype / phenotype	Source or reference
SC5314	*Candida albicans*	Wild-type reference strain	Gillum *et al.* (46)
HUC62	*C. albicans*	CA clinical isolate	Walker *et al.* (42)
HUC65	*C. albicans*	CA clinical isolate	Walker *et al.* (42)
HUC103	*C. albicans*	CA clinical isolate	Nye *et al.* (15)
HUC104-05	*C. albicans*	CA clinical isolate	Nye *et al.* (15)
HUC110	*C. albicans*	CA clinical isolate	Nye *et al.* (15)
HUC110 *mrr1Δ/Δ*	*C. albicans*	Prototrophic nourseothricin sensitive CRISPR deletion mutant of *MRR1* in HUC110 background	This study
HUC117-01	*C. albicans*	CA clinical isolate	Nye *et al.* (15)
HUC119-06	*C. albicans*	CA clinical isolate	Nye *et al.* (15)
HUC123-06	*C. albicans*	CA clinical isolate	Nye *et al.* (15)
HUC125-04	*C. albicans*	CA clinical isolate	Nye *et al.* (15)
HUC135-07	*C. albicans*	CA clinical isolate	Nye *et al.* (15)
O-V0	*C. albicans*	CA clinical isolate	Armbruster *et al.* (16)
J-V10	*Candida dubliniensis*	CA clinical isolate	Armbruster *et al.* (16)
A-V23-2	*Candida orthopsilosis*	CA clinical isolate	Armbruster *et al.* (16)
C-V24	*Candida parapsilosis*	CA clinical isolate	Armbruster *et al.* (16)
M-V4-2	*C. parapsilosis*	CA clinical isolate	Armbruster *et al.* (16)
MYA-3404	*Candida tropicalis*	Wild-type reference strain	Butler *et al.* (116)
HUC98-06	*C. tropicalis*	CA clinical isolate	Nye *et al.* (15)
G-V22	*Clavispora lusitaniae*	CA clinical isolate	Armbruster *et al.* (16)
BG2	*Nakaseomyces glabratus*	Laboratory strain	Cormack & Falkow (117)
HUC124-03	*N. glabratus*	CA clinical isolate	Nye *et al.* (15)
M-V4-1	*N. glabratus*	CA clinical isolate	Armbruster *et al.* (16)
UTI89	*Escherichia coli*	Uropathogenic isolate (cystitis)	Chen *et al.* (118)
JE2	*Staphylococcus aureus*	USA300 derivative, plasmid-cured	Fey *et al.* (92)

Table S2

Table S2 is included as a separate file and includes the full RNA-seq dataset shown in Fig. 3 and Fig. S2.

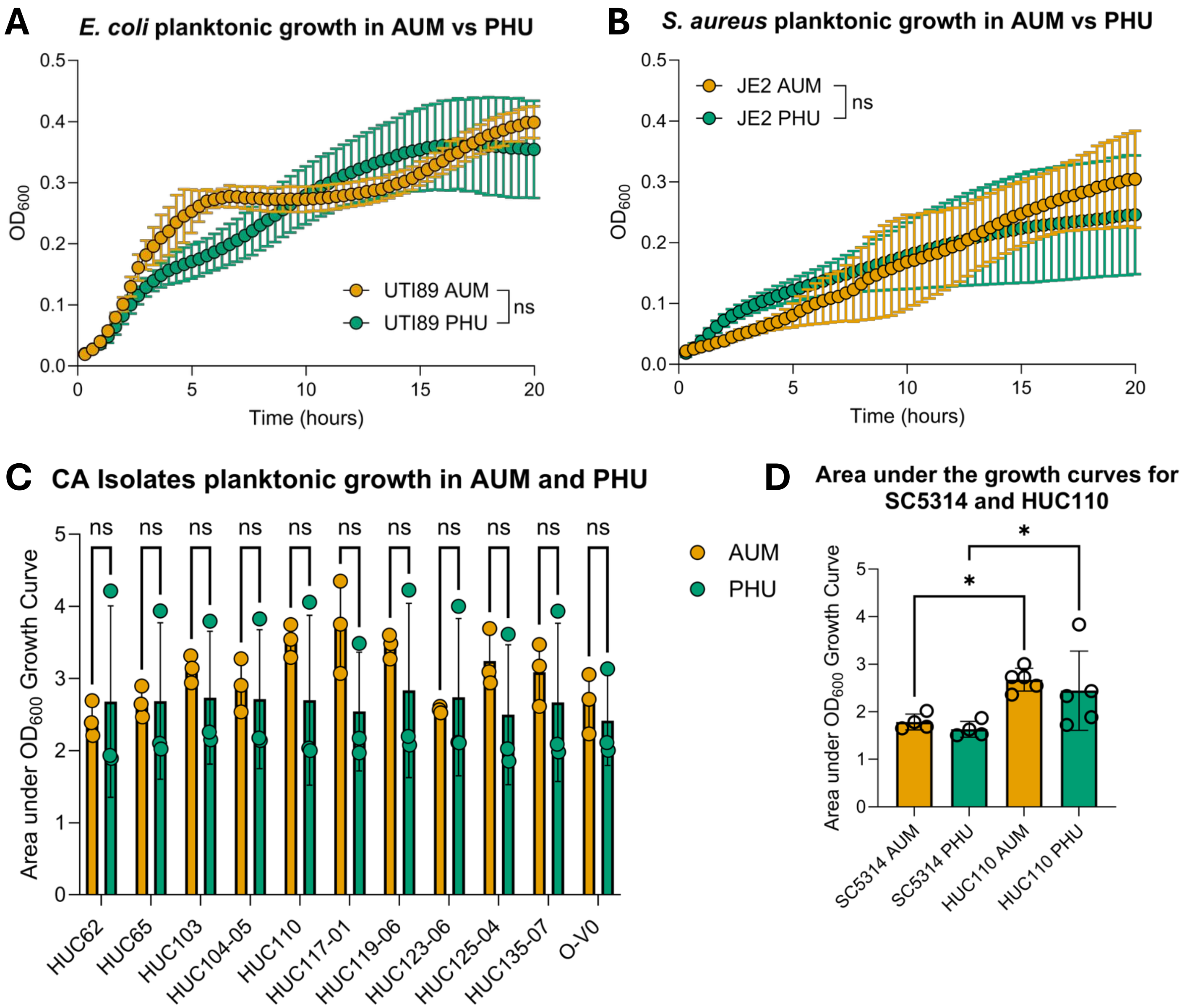

Figure S1: A novel artificial urine media (AUM). (A) Planktonic growth of *Escherichia coli* UTI89 in AUM and pooled human urine (PHU). (B) Planktonic growth of *Staphylococcus aureus* JE2 in AUM and PHU. (C) Comparison of the area under the curve for the panel of 11 *C. albicans* CA isolates grown in AUM and PHU. (D) Comparison of the area under the curve for *C. albicans* SC5314 and HUC110 grown in AUM and PHU. Area under the curve was calculated and compared with a one-way ANOVA test with Sidak's multiple comparisons test; ns *P*-value ≥ 0.05, **P* < 0.05.

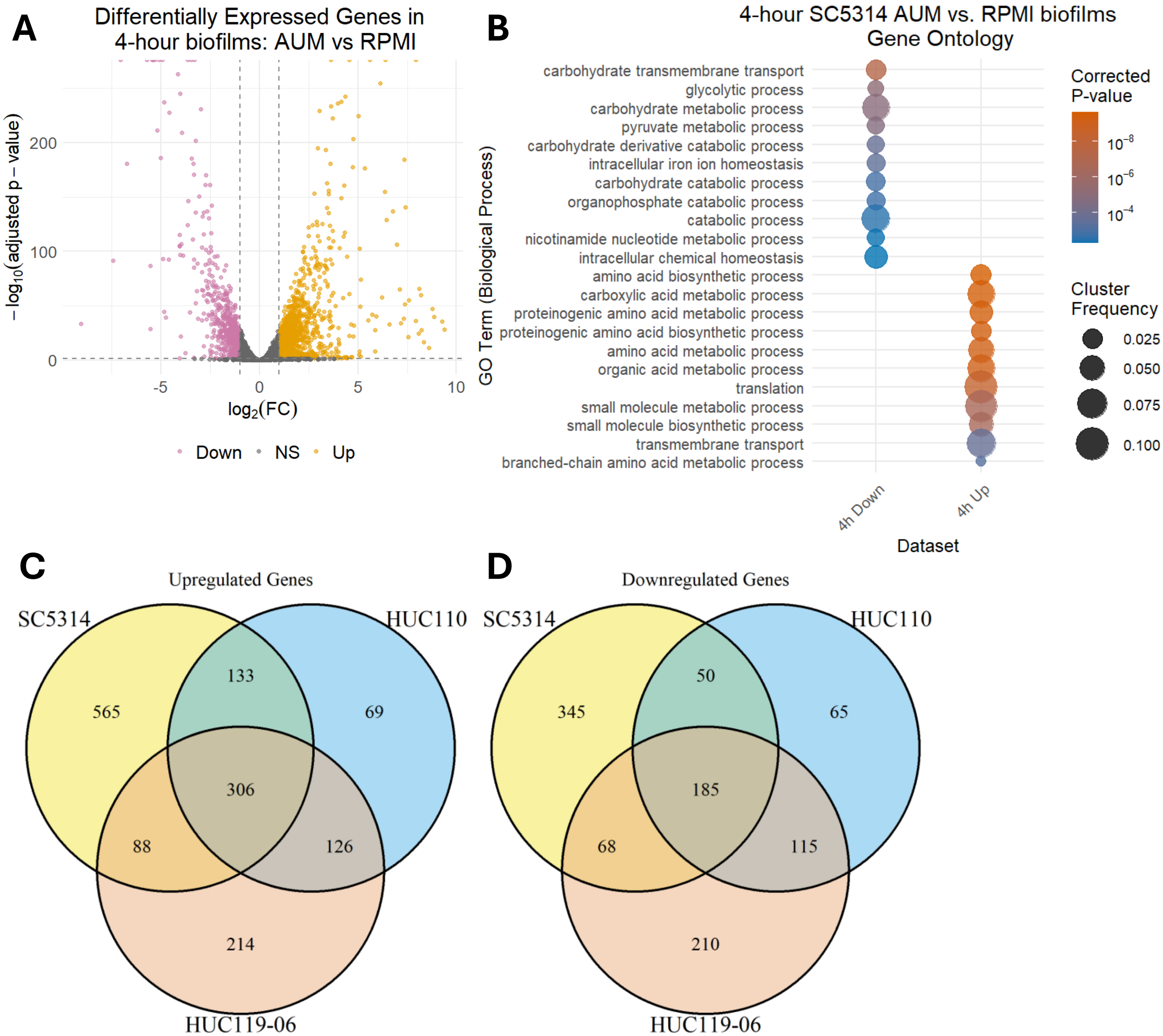

Figure S2: Early artificial urine media (AUM) biofilm development. (A) 1740 genes were differentially expressed in 4-hour biofilms of SC5314 in AUM (Upregulated) vs. RPMI (Downregulated). (B) Gene Ontology analysis of significantly different processes in SC5314 in AUM (vs. RPMI) at 4 hours. (C & D) Comparison of genes significantly (C) upregulated or (D) downregulated in SC5314, HUC110, and HUC119-06 in AUM at 4 hours (all vs. SC5314 in RPMI at 4 hours).

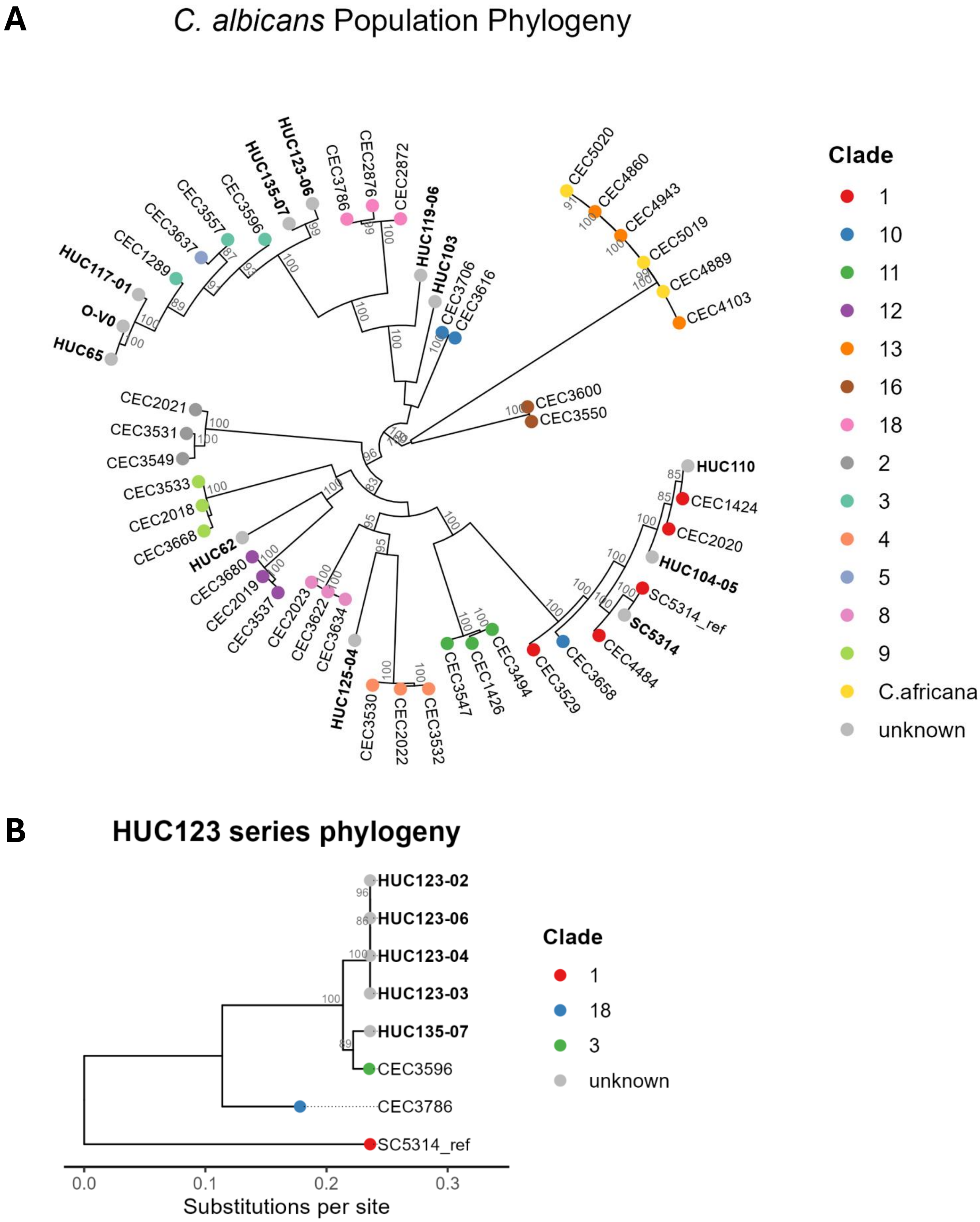

Figure S3: CA Isolates are genetically diverse. (A) Maximum likelihood phylogeny of CA isolates (bold) and a subset of a previously described genome collection (62) spanning the major clades of *Candida albicans*. The tree was constructed using midpoint rooting in IQ-TREE from concatenated genome-wide SNPs called against the haploid SC5314 A22 reference under the GTR+G4 model. The complete dataset contained 356,389 biallelic SNPs. Numbers at nodes indicate ultrafast bootstrap support (only ≥ 80). Branch lengths are proportional to substitutions per site. (B) Subset of total tree examining the four HUC123 series of isolates alongside the most closely related isolates. (A & B) Tip points are color-coded by multi-locus sequence typing clade.

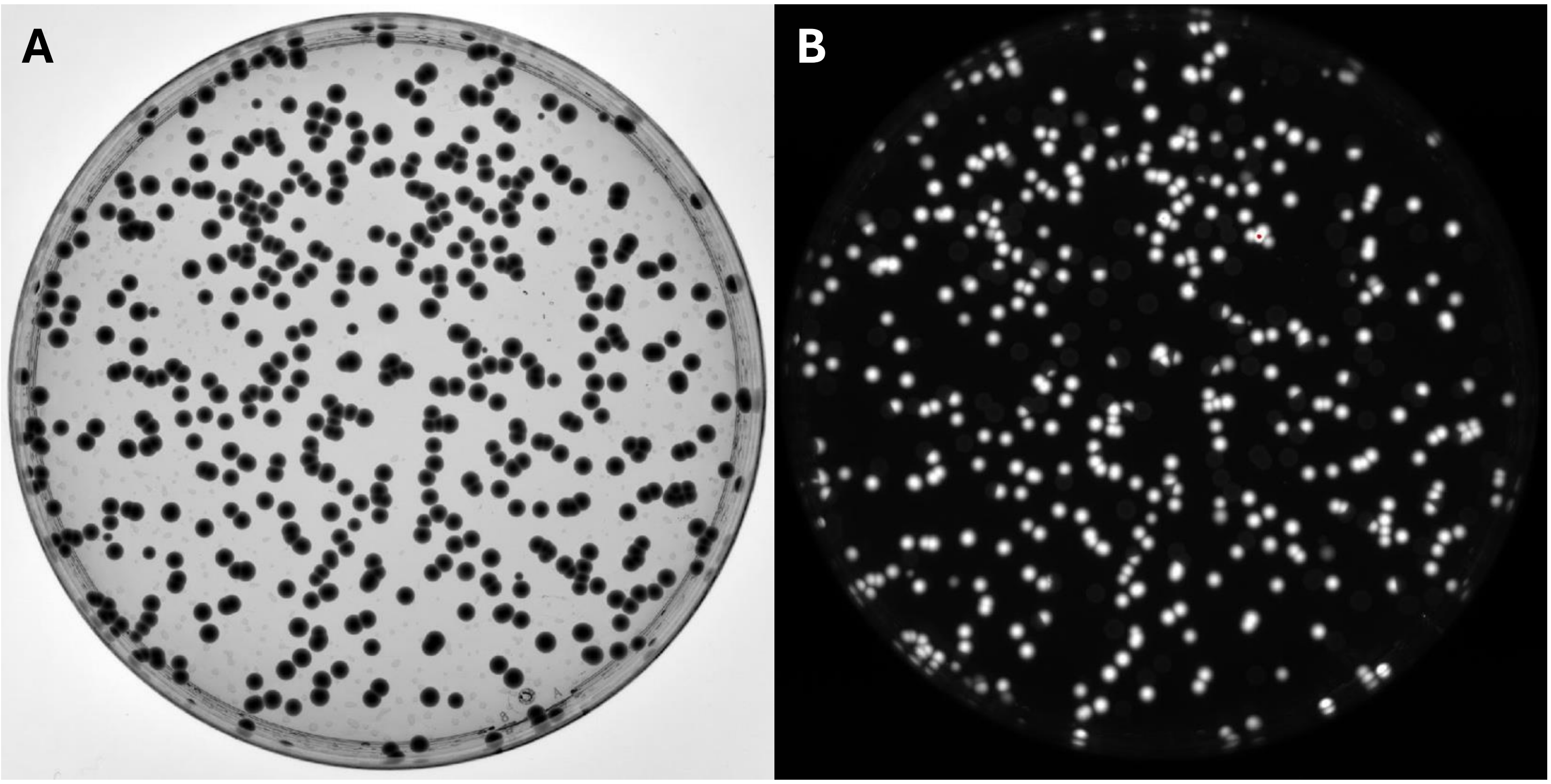

Figure S4: Representative images of a plate from the competition assay. After *in vitro* competition, cells were diluted and plated onto YPD for imaging. (A) A brightfield exposure captures all colonies on the plate, and (B) fluorescent imagining isolates only the WT HUC110 tagged with tdTomato colonies.

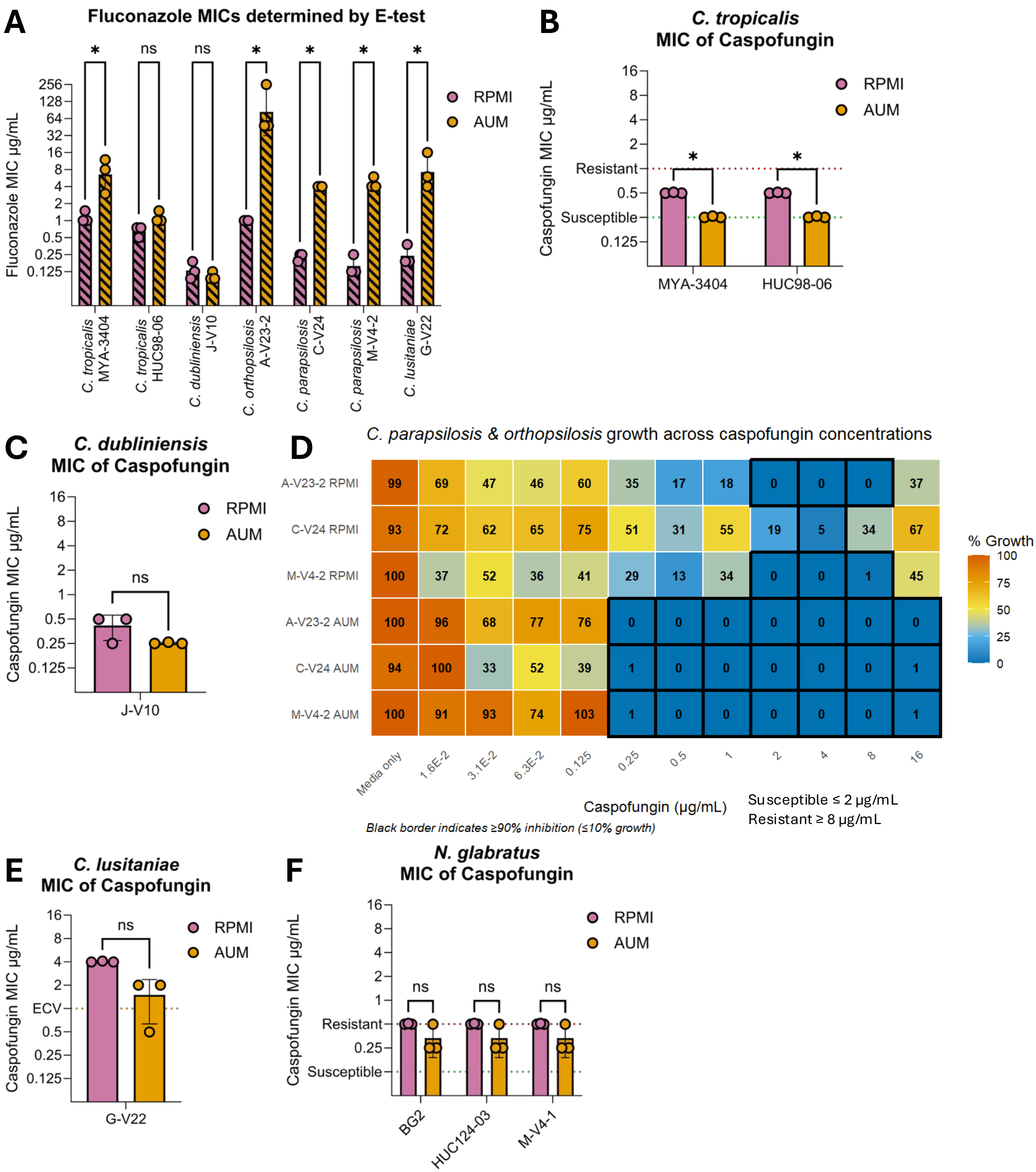

Figure S5: Fluconazole E-test and caspofungin MICs for other fungal catheter-associated isolates. (A) Fluconazole E-tests performed on RPMI and artificial urine media (AUM) plates. (B) Planktonic caspofungin MICs for *Candida tropicalis* isolates. (C) Planktonic caspofungin MICs for *Candida dubliniensis* isolates. (D) Median growth (as a percent vs. untreated) of *Candida orthopsilosis* and *Candida parapsilosis* isolates in a dilution series of caspofungin. (E & F) Planktonic caspofungin MICs of (E) *Clavispora lusitaniae* and (F) *Nakaseomyces glabratus* isolates. For all (A – F) MICs were determined as the amount of drug needed to reduced the OD_{600} by 90% Differences between strains grown in RPMI and AUM were determined using lognormal t-tests (Welch's), and multiple comparisons were calculated using the Holm-Sidak method with an alpha value of 0.05; Adjusted *P*-values: **P* < 0.05.
